# Funny or Angry? Neural Correlates of Individual Differences in Aggressive Humor Processing

**DOI:** 10.3389/fpsyg.2019.01849

**Published:** 2019-08-21

**Authors:** Xiaoping Liu, Yueti Chen, Jianqiao Ge, Lihua Mao

**Affiliations:** ^1^School of Psychological and Cognitive Sciences, Peking University, Beijing, China; ^2^Beijing Key Laboratory of Behavior and Mental Health, Peking University, Beijing, China; ^3^Center for MRI Research, Academy for Advanced Interdisciplinary Studies, Peking University, Beijing, China

**Keywords:** aggressive humor, social adaption, self-cognition, anger control, fMRI

## Abstract

Humor has been a hot topic for social cognition in recent years. The present study focused on the social attribute of humor and showed different stories to participants, which were divided into four types according to the model of humor style, to explore the underlying neural mechanism of point-to-self aggressive humor and how individual differences modulated it. Measuring the degree of anger and funniness, results suggested that aggressive humor helped us in social communication by reducing the degree of anger. The neural activities showed that bilateral temporal lobes and frontal lobes played a synergistic role in the point-to-self aggressive humor processing, while point-to-self non-aggressive humor was dominant in the left-side brain. Results from the region of interest (ROI) analysis showed that the individual differences of the self-control level and the self-construal level may influence the neural processing of point-to-self aggressive humor by modulating the activated levels and patterns of the right inferior orbital frontal gyrus, the right superior temporal lobe, and the right superior frontal lobe.

## Introduction

Humor is common in human society and plays an important role in relieving stress and promoting interpersonal relationship. Martin (2010) was one of the first psychologists who studied humor systematically. He put forward that humor refers to what people said or did anything considered funny and make others laugh, and also refers to the mental process of creating and perceiving things that are funny, and the emotional reaction of appreciating these things. Martin’s explanation of humor focused on the psychological cognitive process and analyzed humor from three key aspects: the source of humor, the process of humor, and the emotional response of humor (Martin, 2010).

### The Psychological Mechanism of Humor

Incongruity-Resolution theory, Cognitive-Emotional theory, and the superiority theory are the psychological mechanisms of humor that are currently recognized by psychologists. Although these theories’ emphases are different, they are not mutually exclusive as they attempted to explain the psychological mechanism of humor from different perspectives.

The Incongruity-Resolution theory (Suls, 1972) stated the mechanism of the source of humor from the perspective of information processing. Sulu’s theory divided the processing of humor into detection and resolution of incongruity. In the process of receiving the setup part of humor stories, individuals would have an expectation about the rest of the stories, but when they received the punchline, they found that the end of the stories did not accord with their expectation, and then it generated an incongruity state. Later, individuals entered the stage of problem-solving in order to find the resolution that made the relationship between punchline and setup logical and reasonable. Finally, a pleasant feeling was experienced due to an effective resolution found after the incongruity state (Suls, 1972).

Different from the Incongruity-Resolution theory, the Cognitive-Emotional theory paid more attention to the process of humor and the emotional response of humor. It divided the processing of humor into two components: cognitive and emotional (Gardner et al., 1975). The comprehension of the humor during the incongruity resolution stage was classified as the cognitive component, while the appreciation (the emotional reaction) of humor after the incongruity resolution stage was classified as the emotional component. The separation of cognitive and emotional components in humor processing was not only the time separation but also the separation for different parts of humor processing. It was extremely important for further studies on humor.

Both the Incongruity-Resolution theory and the Cognitive-Emotional theory tried to explain the psychological mechanism of humor from different aspects of cognitive processing. In comparison, the superiority theory of humor (Wilkins and Eisenbraun, 2009) emphasized the social adaption function of humor. The superiority theory suggested that aggression was a component of humor (Vrticka et al., 2013). The function of humor was to transform the inevitable discomfort about maintaining social order into a more positive emotional experience. In the superiority theory, humor made laughing at others more acceptable for individuals who were laughed at (Wilkins and Eisenbraun, 2009).

### The Neural Mechanism of Humor

Research on the neural mechanism of humor has focused on two important areas: whether there was lateralization in humor processing (e.g., whether there was a dominant hemisphere of the brain in humor processing) and whether the cognitive and emotional components of humor can be supported by neuroscience and what were the specific mechanisms of these two components.

For the lateralization in humor processing, evidences from neuropsychological studies suggested that patients with damage in the right hemisphere of the brain had bigger obstacles to process humor than patients with brain damage in the left hemisphere (Gardner et al., 1975; Bihrle et al., 1986). Making a more detailed division of the diseased brain regions, Shammi and Stuss (1999) indicated that the right frontal lobe played a critical role in the humor integration of the cognitive process and emotional process. However, measuring differences in alpha wave between the left and right hemispheres of the brain during humor processing in electrophysiological studies, researchers found that the differences in alpha wave between the left and right hemispheres were smaller in processing humor than non-humor, suggesting that the left and right hemispheres of the brain were synergistic rather than one of them being dominant in humor processing (Svebak, 1982).

In functional magnetic resonance imaging (fMRI) studies about cognitive and emotional components of humor, researchers usually discussed the mechanism by comparing humor and non-humor materials in various forms. Goel and Dolan (2001) first used event-related fMRI technology to explore the neural mechanism of humor by using semantic and phonological jokes. Study findings indicated that semantic humor processing activated the left middle temporal gyrus, the left inferior temporal gyrus, the right middle temporal gyrus, and cerebellum, while phonological humor processing activated the left inferior temporal gyrus and the left inferior frontal gyrus. By comparing the participants’ ratings of funniness of different kinds of materials, researchers concluded that the brain regions associated with the emotional component of humor processing were the bilateral ventromedial prefrontal cortex and cerebellum in the reward system. To avoid the interference of language processing on humor processing (Bartolo et al., 2006; Samson et al., 2008), used cartoon materials without language information in research and found that the right inferior frontal gyrus, the left superior temporal gyrus, and the middle temporal gyrus, as well as the left cerebellum, were significantly activated, and the amygdala played a key role in the emotional component of humor processing. Another research also used cartoon materials to compare cognitive-conflict materials and humor materials and suggested that the former only included cognitive processing, while the latter activated reward pathways and the brain regions related to cognitive processing (Amir et al., 2013). Having collected the laughter time of participants while watching a humor video, as a standard to define the duration between the cognition processing and the emotion processing, Moran et al. (2004) found that the left inferior frontal gyrus and the middle temporal gyrus were significantly activated in cognitive processing, and the bilateral amygdala and the insula were significantly activated in emotional experience.

Sound, cartoon, and video materials were vivid forms of humor in daily life, but analysis of them were limited due to the simple classification of these materials being “funny” or “unfunny,” and studies using these three kinds of materials didn’t have a clear distinction between cognitive and emotional components. Chan et al. (2012) used three kinds of humor stories as experimental materials: those involved in cognitive processing and emotional processing at the same time, those involved in cognitive processing but no emotional processing (e.g., garden-pathway), and those involved in neither of them. It was better for distinguishing cognitive and emotional components of humor. The region of interest (ROI) analysis found that the bilateral inferior frontal gyrus and the left superior frontal gyrus were related to the comprehension of humor, while the ventral medial prefrontal cortex, the parahippocampal gyrus, and the amygdala were related to the pleasure emotion triggered by humor. Shibata et al. (2014) believed that the humor story materials could maintain the consistency of setup between the experimental group and the control group, and so they used the same paradigm to explore the cognitive and emotional components of humor.

Currently, there is no consensus on the lateralization of brain in humor processing; however, there is evidence supporting the cognitive and emotional components of humor (Gardner et al., 1975; Svebak, 1982; Bihrle et al., 1986; Shammi and Stuss, 1999; Goel and Dolan, 2001; Moran et al., 2004; Bartolo et al., 2006; Franklin and Adams, 2011; Chan et al., 2012; Shibata et al., 2014). The cognitive processing of humor was related to the activation of the left inferior frontal gyrus, bilateral middle temporal gyrus, inferior temporal gyrus, and anterior cingulate gyrus. The emotional processing of humor was related to the activation of the left parietal cortex, right inferior frontal gyrus, insula, and reward circuits including the ventromedial prefrontal cortex, thalamus, and amygdala (Goel and Dolan, 2001; Moran et al., 2004; Bartolo et al., 2006; Chan et al., 2012; Amir et al., 2013; Shibata et al., 2014). It is worth noting that using different paradigms with different forms of experimental materials makes different results.

### The Model of Humor Style and Aggressive Humor

Previous research usually focused on different forms of humor. However, Martin et al. (2003) proposed the model of humor style, which paid more attention to the social interactions among characters. It provides a standard of categorizing humor stimuli. According to the direction of humor and the aggression of the interpersonal relationship between these two dimensions, the model of humor style could divide the verbal humor into point-to-self non-aggressive humor, point-to-self aggressive humor, point-to-others non-aggressive humor, and point-to-others aggressive humor.

Previous behavioral experiments have shown that aggression increased the degree of funniness of humor materials (Epstein and Smith, 1956) and led to decreased levels of pleasure (Willinger et al., 2017). Further exploration showed that the materials with highly aroused aggression made participants feel more pleasure (Strickland, 1959; Dworkin and Efran, 1967). It was a pity that not all studies reached a consensus. Singer et al. (1967) found that highly aroused aggression had no effect on the pleasure feeling after resolution of incongruity during the humor processing. Concluding details of these studies, we found that it was not clear whether aggressive materials were humorous or not and what the direction of humor was, which may affect the degree of funniness of aggressive materials and non-aggressive materials. In addition, Willinger et al. (2017) found that there was a different evaluation of aggression between point-to-self aggression and point-to-others aggression.

To our knowledge, there was no research exploring the neural mechanism of aggressive humor. Aggression was a characteristic of aggressive humor, and provocative aggressive scenes were the most important cause of anger (Anderson and Bushman, 2002). A large number of fMRI studies have focused on the processing mechanism of anger, and their results showed that anger was related to the medial prefrontal cortex, orbital inferior frontal gyrus, cingulate gyrus, and insula (Blair et al., 1999; Phan et al., 2002; Murphy et al., 2003). According to the emotional valence hypothesis, the right hemisphere is responsible for the emotional processing of anger, sadness, nausea, and other negative emotions, while the left hemisphere is responsible for the emotional processing of pleasure, surprise, and other positive emotions (Davidson, 1992). When the pleasure emotion and angry emotion existed at the same time, the activated brain regions contained not only those related to these two emotions, but also those associated with emotional conflict, such as the right dorsolateral superior frontal gyrus, middle temporal gyrus, and superior temporal gyrus, which were different from those activated by the cognitive conflict (Wittfoth et al., 2009). Since there was a conflict between the pleasure caused by humor and the anger caused by aggression, the emotional response caused by aggressive humor would be complex.

It was possible that there would be great individual differences in aggressive humor processing because of its complex emotional processing and high-level cognitive function. Previous study paid attention to the correlates of personality traits and negative emotional states on humor processing, rather than the correlates of individual differences in social interpersonal communication on it (Vrticka et al., 2013; Sara et al., 2018).

Tangney et al. (2004) proposed a scale measuring the self-control level of individuals and found that individuals with high self-control had better mental health, higher self-esteem, better social skills and abilities, as well as more rational emotion management. The processing of aggressive humor must involve emotion management, especially anger control. It was reasonable to believe that individuals with different self-control levels would have differences in the aggressive humor processing.

Concentrating on the direction of humor, Feng et al. (2014) found that the right frontal lobe associated with Theory of Mind (ToM) was important in the point-to-others humor processing. Regardless of being point-to-self or point-to-others, the neural network of the humor processing was associated with the neural network of the self-cognition processing (Van Overwalle and Baetens, 2009; Abu-Akel and Shamay-Tsoory, 2011; Feng et al., 2014), which involved the right prefrontal lobe and the right temporal lobe (Fink et al., 1996; Craik et al., 1999; Keenan et al., 2000a). The concept of self-construal was referred to people’s tendency of self-cognition (Markus and Kitayama, 1991). In the meantime, self-construal was supported to associate with the emotional experience. For the individuals with interdependent self-construal, it was more likely to suppress the emotional response associated with themselves for protecting the collective interests, while the independent self-construal group was more dominated by their own emotion (Lutz, 2011). Involving the self-cognition and the complex emotional control, the aggressive humor was likely to be affected by self-construal.

Both self-control and self-construal are important aspects in social psychology, and the main aim of this study was the social role of humor. The present research would concern the effect of the self-control level and the self-construal level on the aggressive humor processing.

### The Present Study

Since previous studies rarely focused on the important social attributes of humor and there was no detailed discussion of the neural mechanism of humor on the direction and the aggression of these two dimensions in the model of humor style, the present study focuses on the (neural) mechanism of aggressive humor based on its social adaptive function, and the correlation of individual differences (self-control and self-construal) with it.

According to the results of Feng et al.’s (2014) study that the point-to-others humor induced more ToM processing than the point-to-self humor, we selected the point-to-self humor stories as experimental materials to avoid the interference of ToM.

The present study formulated three hypotheses:

*Hypothesis 1:* aggression would reduce the degree of funniness of aggressive humor stories since the aggressive humor was point-to-self; however, humor would reduce the degree of anger of aggressive humor stories, which showed the social function of humor in promoting people’s social relationship.
*Hypothesis 2:* for the neural mechanism of aggressive humor, the point-to-self aggressive humor would show the brain region associated with cognitive process and reward pathways activated, which were the same activated regions in non-aggressive humor, and there would be some brain regions associated with the anger control and the self-cognition activated by aggressive humor especially; the lateralization of humor processing was related whether the humor was aggressive or not according to the emotional value theory.
*Hypothesis 3:* the brain regions activated by the point-to-self aggressive humor specially would correlate with the self-control level and the self-construal level. The high self-control group and the low self-control group would show different rating patterns and different activation patterns. The interdependent self-construal group would show different rating patterns and different activation patterns from the independent self-construal group.

## Materials and Methods

### Participants

Twenty-seven undergraduate students from a large university in Beijing, China, participated in this study. Two of them did not complete the experiment. The final sample consisted of a total of 25 participants (11 women; age range: 18–25 years old). All participants were native Chinese speakers, were right-handed, had normal vision or corrected-to-normal vision, had no reading disorder, and were given written informed consent before the start of the experiment. The study was approved by the Committee for Protecting Human and Animal Subjects at the School of Psychological and Cognitive Sciences of Peking University, China. All subjects gave written informed consent in accordance with the Declaration of Helsinki.

### Materials

Based on the operational definition of “verbal humor containing social context” (Chan et al., 2012), 80 humorous stories without sexual content or social taboo were collected as experimental materials. Each humorous story consisted of two components: the setup and the punchline. Half of the stories were aggressive stories (i.e., the literal meaning of the punchline in dialogue was derogatory to others) and the others were not aggressive (i.e., the literal meaning of the punchline in dialogue was not derogatory to others). Then, we added some reasonable scenes in dialogue for the stories without clear social context, simplified the length of stories, and used fictitious names (i.e., “Xiao Wang,” “Xiao Li”) or their job titles to replace the real names in stories. Later, 25 aggressive and 25 non-aggressive humorous stories were selected after we filtered out some humorous stories with unclear boundaries between aggressive and non-aggressive, and the punchline in every story was rewritten to produce its corresponding non-humorous version. Furthermore, 100 experimental materials were modified to the conversations between participant and the other people, and the last sentences were pointed to the self (people who read stories).

Finally, there were four types of experimental materials (25 materials in each condition) in the study: point-to-self aggressive humorous stories (AH, the punchline contained derogatory meaning to participants and it was funny), aggressive non-humorous materials (AnH, the punchline contained derogatory meaning to participants but it wasn’t funny), non-aggressive humorous materials (nAH, the punchline didn’t contain derogatory meaning to participants but it was funny), and non-aggressive non-humorous materials (nAnH, the punchline didn’t contain derogatory meaning to participants and it wasn’t funny). All stories can be found in the Supplementary Data Sheet. The inter-rater reliability of four types of materials in our study were respectively 0.951, 0.947, 0.850, and 0.838.

Take specific examples of four types of materials as reference:

AH condition and AnH condition

You buy a fish, ask your roommate to cook dinner, and go to a movie by yourself. Your roommate wants to go to the movie with you, but you refuse:

“You cook the fish at home and when I come back from the movie, I can tell you what content it is.”

When you come home and want to eat the fish, your roommate says to you:

(AH condition) “I have eaten the fish. I can tell you what taste it was.”

(AnH condition) “Why should I cook fish for you when you’re so selfish?”

nAH condition and nAnH condition

You’re a student assistant at the weather bureau. One day the meteorologist tells you to write down the weather forecast for Sunday: it is cloudy in the morning and rainy in the afternoon.

“I’m going to the amusement park on Sunday afternoon,” you say with a sigh.

The meteorologist said sympathetically:

(nAH condition) “Cut out the “rainy,” then.”

(nAnH condition) “Change the date to the amusement park, then.”

In these stories, the initiator of humor and aggression is the other people, the receiver is “you,” and it is the point-to-self aggressive humor. The “point-to-self” in the current experiment was based on the humor receiver, different from the definition of Martin et al. (2003).

### Procedure

A 2 (aggressive vs. non-aggressive) × 2 (humorous vs. non-humorous) in-group design was used. Degree ratings of anger and funniness of stories and the level of neural activity in four conditions were recorded.

One hundred stories (25 AH, 25 nAH, 25 AnH, and 25 nAnH) were presented randomly for each participant in an event-related fMRI paradigm. The entire fMRI experiment was divided into five runs. Each run included 20 trials (5 AH, 5 nAH, 5 AnH, and 5 nAnH) and lasts 8–9 min. Rest was allowed for a period of time between two runs. The total scanning lasted about 40–45 min.

**FIGURE 1:**
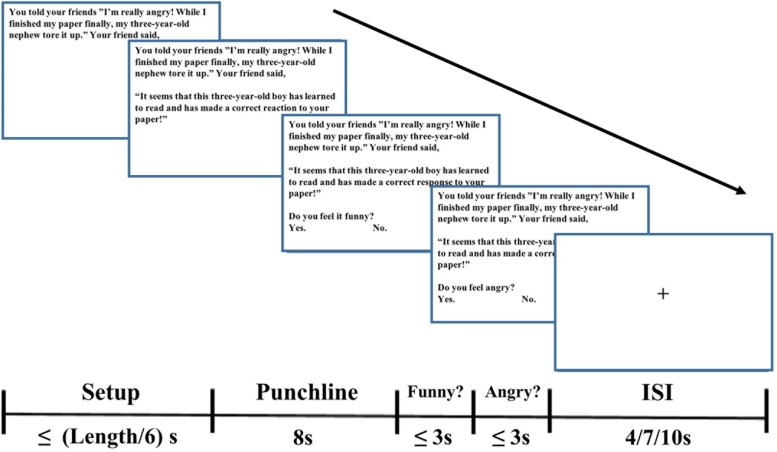
The experimental procedure.

In each trial (Figure 1), participants were first shown the fixation in the center of the screen for a jittered inter-stimulus interval (ISI), which was randomly varied among 4, 7, and 10 s and counterbalanced across all conditions. Subsequently, the setup was shown. To ensure that participants can finish reading of the setup part, participants could press the button to enter the next part after their reading, but we controlled the maximum reading time, which was the number of words in setup divided by 6 s. In the interview after the experiment, we confirmed that all participants could finish reading before the setup part automatically entered the next part. After participants pressed the button, the punchline part appeared in the middle of the screen and lasted for 8 s. And then the questions “do you feel it funny?” and “do you feel angry?” appeared at the bottom of the screen one after another. Considering the time in fMRI experiment, ratings to these two questions were on two points. Participants were instructed to use a keypad to make a choice, “funny”/”not funny” or “angry”/”not angry.” The first question disappeared after participants made a choice on it and then, the second question appeared. If the duration of each question was more than 3 s, the program would automatically enter the next part and recorded the answer as the missing value.

One week after the fMRI experiment, participants did some re-ratings to the similar materials on a nine-point scale and filled in the self-control scale and the self-construal scale.

### Measurements

#### Brief Self-Control Scale

The Brief Self-Control Scale developed by Tangney et al. (2004) has 13 items. The Chinese version we used was from Dang et al. (2017). Participants would choose the level to which they agree with each item on a scale from 1 (= strongly disagree) to 7 (= strongly agree). The higher score means the higher level of self-control. The internal consistency of this scale in the current study was 0.701.

#### Self-Construal Scale

Singelis (1994) developed a Self-Construal Scale consisting of 24 items to measure the individual differences in independent and interdependent self-construal with a seven-point scale. We used its Chinese version in the current study (Ma et al., 2014; Wang et al., 2015; Liu et al., 2018). Participants would choose the level to which they agree with each item on a scale from 1 (= strongly disagree) to 7 (= strongly agree). Consistent with previous studies, the intercultural value tendency was obtained by subtracting the score of 12 interdependent self-construal items from the score of 12 independent self-construal items. The higher the score was, the stronger the intercultural value tendency was. The internal consistency of this scale in the current study was 0.569.

### Image Acquisition

The functional images were acquired on a 3T SIEMENS Prisma scanner with a 64-channel head coil at the Peking University Magnetic Resonance Imaging Center. High-resolution T1-weighted structural images were acquired using the MPRAGE sequence with 0.5 × 0.5 × 1.0 mm in-plane spatial resolution (512 × 512 × 256 matrix, TR = 2,530 ms, TI = 1,100 ms, TE = 2.98 ms, FOV = 256 mm, flip angle = 7°, and thickness = 1 mm). Functional images were obtained using gradient-echo echo-planar imaging (GE-EPI) sequence (64 × 64 matrix, TR = 2,000 ms, TE = 30.0 ms, FOV = 224 mm, flip angle = 90°) with 3.5 × 3.5 × 3.5 mm in-plane spatial resolution in five runs. Each run was for 8–9 min. Each brain image consisted of 33 axial slices with a thickness of 3.5 mm. The program was compiled by Matlab, and the stimuli were projected onto the mirror placed in the MRI scanner.

### Image Analysis

All fMRI data were analyzed by Statistical Parametric Mapping software (SPM8; Wellcome Department of Cognitive Neurology, London, United Kingdom). The image data were formatted into an Analyze (HDR/img) format that would be recognized by SPM8. Then, we used the data of each participant in the fine structure scanning process and motion parameters generated in the head movement correction process to correct the errors caused by the time difference of scanning at different layers and the tiny errors caused by head movement. After all the image sequences were normalized, they were smoothed by a three-dimensional Gaussian function to improve SNR and then they were put into the next analysis. When the preprocessing was done, we checked the head movement data and made sure the displacement was no more than 1.5 mm and the rotation was no more than 1.5° in each direction of every participant.

In the first-level statistical analysis, we used a generalized event-related design model to process the data of each participant. The punchlines in the four kinds of materials were the four experiment conditions (for 8 s in each trial) and six motion parameters of the head were put into the model as covariates. In addition, a dummy variable was used to encode each of the five scan sections. The hemodynamic response function (HRF) was used to convolve the events in different conditions. We used the data in the humorous condition to subtract the data in the non-humorous condition to show which brain regions were activated differently between the humorous condition and the non-humorous condition, and did the same step between the aggressive condition and the non-aggressive condition. On the basis of these analyses, we used the (1, -1, -1, 1) model to define the interaction between these two factors: humor and aggression, and compared differences of the neural response between AH–AnH and nAH–nAnH.

Later, we put the parameter estimation of each subject in the first-level analysis (single subject analyses) into the second-level analysis (group analysis) and analyzed the random effects using *t* test.

One-sample *t* test was used to examine the activated differences between different types of stories voxel by voxel. The activated threshold of ROIs was set at a voxel-wise uncorrected *p* < 0.001 at the cluster level of brain regions. Then, we combined statistical results of 25 participants for the random effect group analysis and defined the brain regions that were more than 10 contiguous voxels with uncorrected *p* < 0.001 as the activated brain regions.

In addition, the center of activated brain regions with uncorrected *p* < 0.001 in the interactional effect or the main effect would be the center of ROIs, which was 6 mm (Feng et al., 2014). A repeated analysis of variance (ANOVA) was used to find out differences of the activated level among different conditions. Finally, effects of the self-control and the self-construal on these differences were investigated.

## Results

### Individual Difference Measurement

#### Self-Control

The mean score on the self-control scale of 25 participants was 49.56 ± 8.61 and ranged from 28 to 62. There were five participants whose scores were 49, which was the median. The participants were divided into the high self-control group (scores were 50 and above, *n* = 10) and the low self-control group (scores were below 50, *n* = 15). The scores of the high self-control group were significantly higher than those of the low self-control group [*t*(23) = 6.564, *p* < 0.001, *d* = 2.77], indicating that the grouping was effective.

#### Self-Construal

The mean score on the self-control scale of 25 participants was 5.16 ± 10.27 and ranged from −17 to 30. There were two participants whose score was 2, which was the median. According to their scores, the participants were classified into the interdependent self-construal group (scores were 2 and above, *n* = 13) or the independent self-construal group (scores were below 2, *n* = 12). The scores of the interdependent self-construal group were significantly higher than those of the independent self-construal group [*t*(23) = −5.806, *p* < 0.001, *d* = −2.421], indicating that the grouping was effective.

### Behavioral Results

#### Rating Results in fMRI Experiment Scan

The participants were requested to rate the degree of funniness and anger of stories on a two-point scale (funny/not funny, angry/not angry) during the scanning procedure. The choice of “funny” or “angry” would be scored four points and the choice of “not funny” or “not angry” would be scored zero points. If the participant was too hesitant and missed the decision time, it would be scored two points, which happened rarely. To each participant, we summed up the rating scores of 25 trials of each condition. The score range was 0–100. The higher the score was, the funnier the participant thought the material was or the angrier the participant felt. The descriptive statistics are depicted in Table 1.

**TABLE 1:** Means (standard error) of the degree of funniness and anger rating.

	**AH**	**AnH**	**nAH**	**nAnH**
Degree of funniness	69.36 (3.34)	7.60 (2.05)	77.44 (2.37)	11.04 (1.99)
Degree of anger	36.48 (3.64)	71.92 (4.19)	6.08 (0.95)	11.84 (1.23)

**FIGURE 2:**
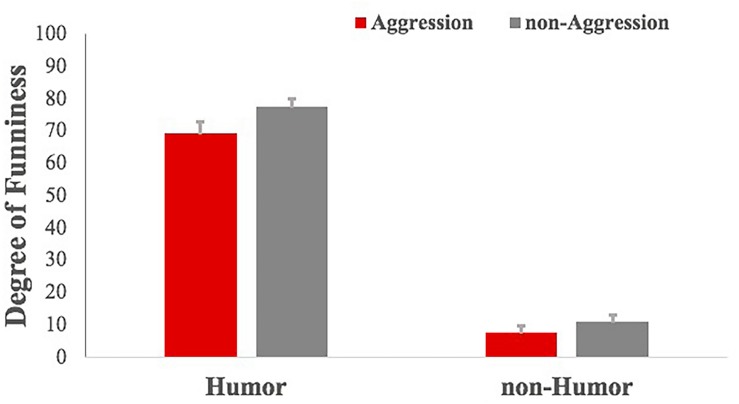
Comparison of the degree of funniness of experimental materials under the four conditions.

**FIGURE 3:**
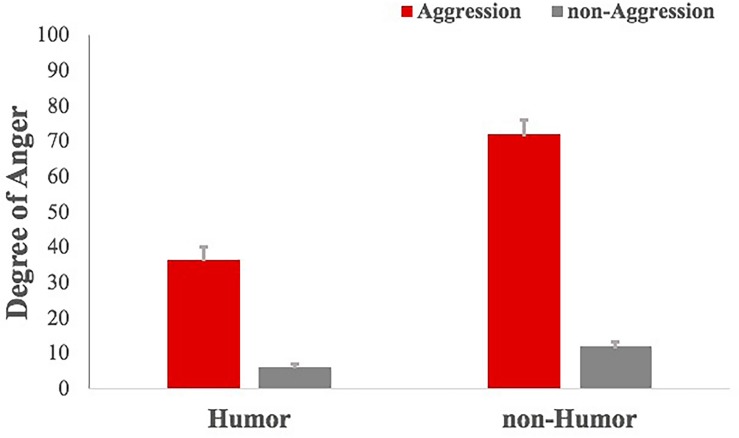
Comparison of the degree of anger of experimental materials under the four conditions.

For the degree of funniness, results of repeated-measures ANOVA showed that aggression had a significant effect on reducing the degree of funniness of stories, *F*(1, 23) = 15.074, *p* < 0.01, η^2^ = 0.386, power = 0.961, and humor improved the degree of funniness of stories significantly, *F*(1,23) = 362.858, *p* < 0.001, η^2^ = 0.938, power = 1.000 (Figure 2). The interaction between humor and aggression, *F*(1,23) = 2.747, *p* = 0.110, was not significant. For the degree of anger (Figure 3), results of repeated-measures ANOVA showed that aggression had a significant impact on improving it, *F*(1,23) = 156.981, *p* < 0.001, η^2^ = 0.867, power = 1.000, and humor reduced the degree of anger of stories significantly, *F*(1,23) = 104.01, *p* < 0.001, η^2^ = 0.813, power = 1.000. The interaction between humor and aggression, *F*(1,23) = 67.028, *p* < 0.001, η^2^ = 0.736, power = 1.000, was significant. Specifically, there was significantly greater difference of the degree of anger between AnH and AH, *F*(1,23) = 100.157, *p* < 0.001, η^2^ = 0.807, power = 1.000, and smaller difference of the degree of anger between nAnH and nAH, *F*(1,23) = 15.143, *p* < 0.01, η^2^ = 0.387, power = 0.962.

#### Re-rating Results and Individual Difference Measurement

The advantage of re-rating was obvious: because of the sufficient time, we changed the two-point scale to a nine-point scale and participants could make a more detailed and stable evaluation than two dimensions (funny and angry) of materials. To avoid the difference caused by repeated reading, the retest was scheduled 1 week after the fMRI scanning. All scores were adjusted to a linear score of 0–100 points and statistically analyzed. The descriptive statistics were depicted in Table 2.

**TABLE 2:** Means (standard error) of the degree of funniness and anger re-rating.

	**AH**	**AnH**	**nAH**	**nAnH**
Degree of funniness	58.62 (3.25)	8.72 (1.35)	68.11 (2.33)	13.24 (1.71)
Degree of anger	45.42 (3.02)	69.75 (2.42)	17.42 (1.78)	18.75 (1.12)

The self-control and the self-construal were the between-group factors, and humor and aggression were within-group factors in a mixed ANOVA.

**FIGURE 4:**
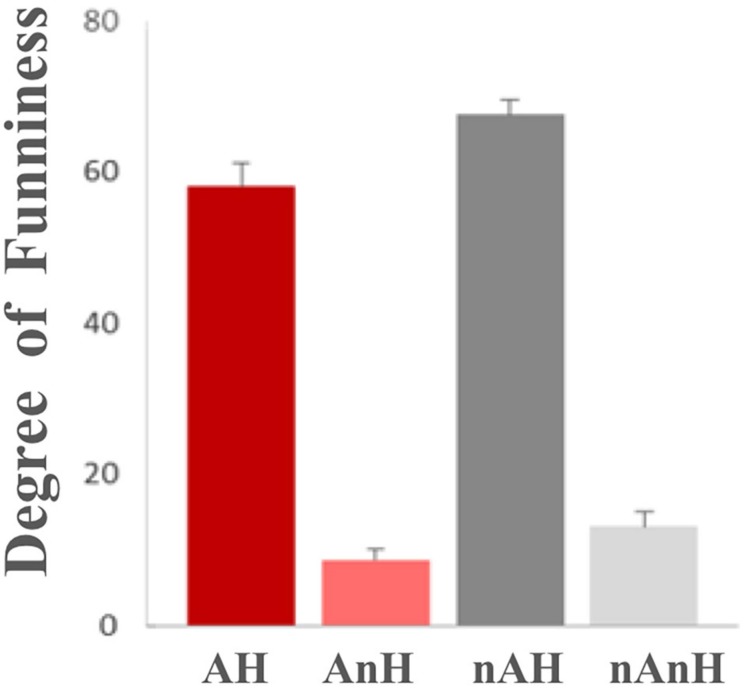
Comparison of the re-rating degree of funniness of experimental materials under the four conditions.

For the degree of funniness, the main effect of aggression was significant (Figure 4), *F*(1,23) = 31.842, *p* < 0.001, η^2^ = 0.581, power = 1.000, which showed that aggression reduced the degree of funniness of stories. The main effect of humor was significant, *F*(1,23) = 348.018, *p* < 0.0001, η^2^ = 0.938, power = 1.000, which showed that humor improved the degree of funniness of stories. The interaction between humor and aggression was significant, *F*(1,23) = 4.906, *p* = 0.037, η^2^ = 0.176, power = 0.564. The group effect of neither self-control, *F*(1,23) = 1.183, *p* = 0.288, nor self-construal, *F*(1,23) = 0.857, *p* = 0.364, was not significant.

**FIGURE 5:**
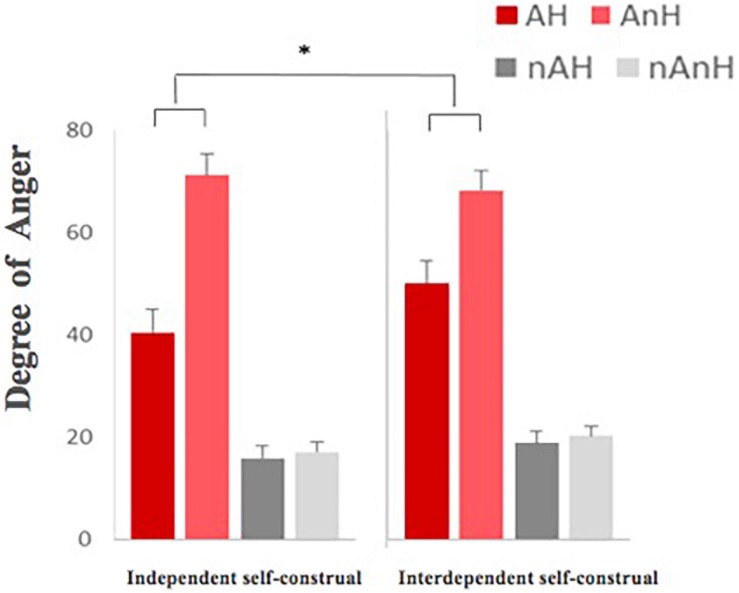
The re-rating degree of anger of stories in four different conditions between independent self-group and interdependent self-group (^∗^ means *p* < 0.05, the 95% confidence interval).

For the degree of anger, the main effect of aggression was significant, *F*(1,23) = 195.508, *p* < 0.001, η^2^ = 0.895, power = 1.000, which showed that aggression improved the degree of anger. The main effect of humor was significant, *F*(1,23) = 48.192, *p* < 0.001, η^2^ = 0.677, power = 1.000, showing that humor reduced the degree of anger. The interaction between humor and aggression was significant, *F*(1,23) = 57.304, *p* < 0.001, η^2^ = 0.714, power = 1.000. Although the group effect of neither self-control, *F*(1,23) = 1.249, *p* = 0.275, nor self-construal, *F*(1,23) = 0.972, *p* = 0.334, was not significant, the interaction among self-construal, humor, and aggression showed significant (Figure 5), which revealed that the differences between AH and AnH in the independent self-construal group were significantly larger than the ones in the interdependent self-construal group, *F*(1,23) = 5.809, *p* = 0.024, η^2^ = 0.202, power = 0.636.

### fMRI Results

#### Neural Mechanism of Aggressive Humor

##### The main effect and the interaction of aggression and humor

**FIGURE 6:**
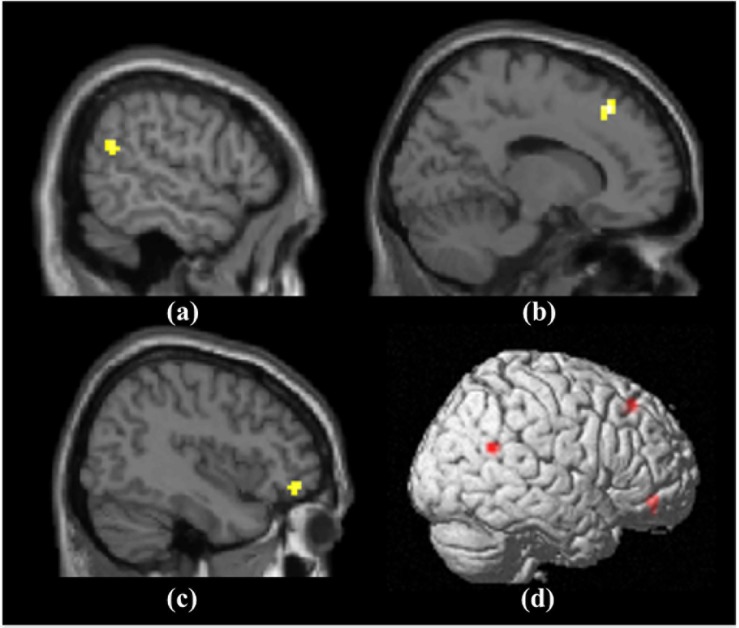
The activated brain region in the interaction between aggression and humor: **(a)** Right superior temporal gyrus; **(b)** Right dorsolateral superior frontal gyrus/medial superior frontal gyrus; **(c)** Right dorsolateral superior frontal gyrus/medial superior frontal gyrus; **(d)** Three activated brain regions on the right side.

The interaction between humor and aggression was significant. Five brain regions activated (or inhibited) significantly after the neural data of the humorous condition were subtracted to those of the non-humorous condition, and six brain regions activated (or inhibited) significantly when the neural data of aggression condition were subtracted to those of the non-aggression condition; when compared with the AH–AnH condition, the right orbital inferior frontal gyrus, right dorsolateral superior frontal gyrus/medial superior frontal gyrus, and right superior temporal gyrus (Figure 6) showed significantly greater activation in the nAH–nAnH condition (Table 3), and later we would combine the individual difference measurement results of self-control and self-construal to ensure the role of these three brain regions in the processing of aggressive humor.

**TABLE 3:** Main effects and interactions between aggression and humor activate brain regions.

	**Region**	**MNI coordinates**	***Z* value**	**Voxels**
Main effect of humor	Left medial temporal gyrus	[−39 −48 21]	6.6128	563
	Right parietal cortex	[12 −75 −6]	–5.2079	71
	Left medial prefrontal cortex	[−45 39 3]	5.7428	293
	Right medial prefrontal cortex	[51 27 9]	5.6522	146
	Left superior frontal gyrus	[−30 6 39]	4.6088	212
Main effect of aggression	Left spindle	[−30 −42 −15]	–5.3292	52
	Left medial temporal gyrus	[−60 −54 −6]	–4.9807	66
	Right parietal cortex	[3 −75 0]	–4.2795	24
	Left limbic system	[−15 −51 6]	–4.4447	21
	Right medial superior frontal gyrus	[12 69 9]	4.37	27
	Medial superior frontal gyrus	[3 57 30]	4.5621	104
Interaction	Right orbital inferior frontal gyrus	[39 45 −15]	3.87	13
	Right dorsolateral superior frontal gyrus/medial superior frontal gyrus	[15 30 48]	5.1175	25
	Right superior temporal gyrus	[60 −54 21]	3.944	12

##### The simple main effects

**FIGURE 7:**
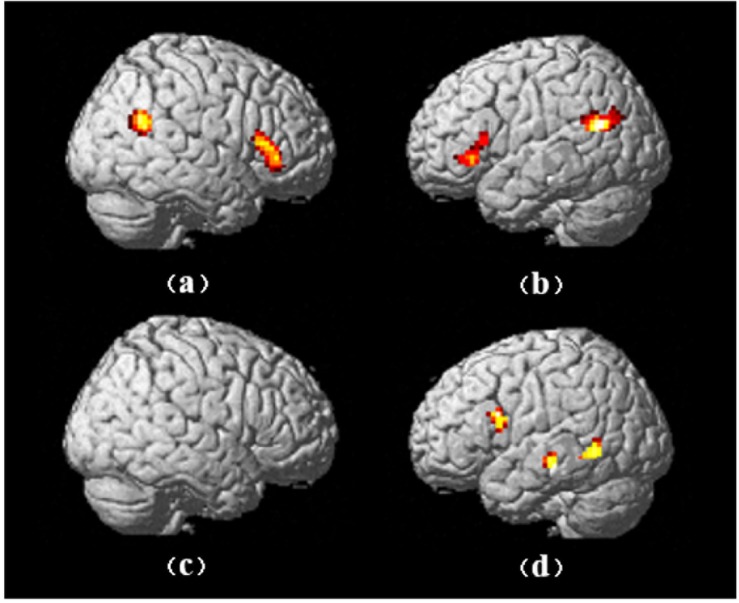
The activated brain regions in the main effects of aggression and humor: **(a)** the right-side brain activations in AH-AnH; **(b)** the left-side brain activations in AH-AnH; **(c)** the right-side brain activations in nAH-nAnH; **(d)** the left-side brain activations in nAH-nAnH.

Based on a whole-brain analysis, the simple main effect analysis showed four significantly activated regions (Figure 7) in the AH–AnH condition (Table 4) and three significantly activated regions in the nAH–nAnH condition (Table 5).

**TABLE 4:** The brain regions activated in AH-AnH condition.

**Region**	**MNI coordinates**	***Z* value**	**Voxels**
Right triangle inferior frontal gyrus/orbital inferior frontal gyrus	[48 33 0]	5.4069	114
Left triangle inferior frontal gyrus/orbital inferior frontal gyrus	[−51 18 12]	4.2051	74
Right angular gyrus/superior temporal gyrus/middle temporal gyrus	[57 −54 24]	5.1816	90
Left medial temporal gyrus/angular gyrus	[−45 −57 21]	4.8401	164

**TABLE 5:** The brain regions activated in nAH-nAnH condition.

**Region**	**MNI coordinates**	***Z* value**	**Voxels**
Left medial temporal gyrus	[−51 −24 −9]	4.5874	35
Left medial temporal gyrus/inferior temporal gyrus	[−54 −54 0]	4.6538	39
Inferior insular gyrus/inferior triangular gyrus	[−45 9 21]	4.2248	51

#### The Regulating Effects of Individual Differences on Aggressive Humor Processing

##### The regulating effect of self-control on the right orbital inferior frontal gyrus

A mixed ANOVA was used: the beta values of fMRI in ROI in four conditions, which was a 6-mm sphere with the center [39, 45, -15], were the dependent variables, humor and aggression were the in-group independent variables, and the self-control was the between-group independent variable. The main effect of group showed greater activation in the high self-control group than the low self-control group in this area, *F*(1,23) = 7.006, *p* = 0.014, η^2^ = 0.233, power = 0.717. The main effect of aggression, *F*(1,23) = 0.141, *p* = 0.711, was not significant, but the interaction between aggression and self-control, *F*(1,23) = 7.846, *p* = 0.010, η^2^ = 0.253, power = 0.765, was significant. The main effect of humor, *F*(1,23) = 3.879, *p* = 0.061, was not significant, and the interaction between humor and self-control, *F*(1,23) = 0.134, *p* = 0.718, was not significant. The interaction between aggression and humor, *F*(1,23) = 6.376, *p* = 0.019, η^2^ = 0.217, power = 0.677, was significant, and the interaction among self-control, aggression, and humor, *F*(1,23) = 3.306, *p* = 0.082, was not significant.

A further test revealed greater activation in the high self-control group than the low self-control group under the aggression condition, *F*(1,23) = 11.121, *p* = 0.003, η^2^ = 0.326, power = 0.891, while there was no significant difference between two groups under the non-aggression condition, *F*(1,23) = 2.673, *p* = 0.116.

**FIGURE 8:**
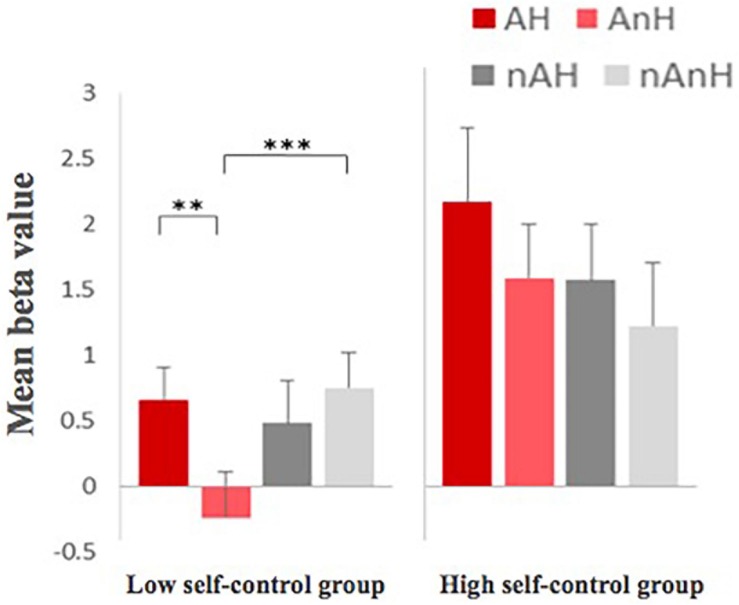
Different activation patterns of the right orbital inferior frontal gyrus [39, 45, −15] between the high self-control group and the low self-control group (^∗∗^ means *p* < 0.01, and ^∗∗∗^ means *p* < 0.001, the 95% confidence interval).

The activation patterns of the brain region in the high self-control group and the low self-control group were significantly different under four conditions (Figure 8). For the low self-control group, the main effect of aggression, *F*(1,14) = 6.527, *p* = 0.023, η^2^ = 0.318, power = 0.662, was significant, showing greater activation in the non-aggression condition. The main effect of humor, *F*(1,14) = 5.078, *p* = 0.041, η^2^ = 0.266, power = 0.555, was significant, showing greater activation in the humorous condition. The interaction between humor and aggression, *F*(1,14) = 14.895, *p* = 0.002, η^2^ = 0.515, power = 0.948, was significant, which showed greater activation in the AH condition than in the AnH condition, *F*(1,14) = 14.210, *p* = 0.002, η^2^ = 0.504, power = 0.938, while no significant differences between nAH and nAnH, *F*(1,14) = 2.533, *p* = 0.134, and showed the lower activation in AnH than in nAnH, *F*(1,14) = 28.831, *p* < 0.001, η^2^ = 0.673, power = 0.999, while no significant differences between AH and nAH, *F*(1,14) = 0.731, *p* = 0.407. For the high self-control group, there was no significant main effect of aggression, *F*(1,9) = 2.504, *p* = 0.148, main effect of humor, *F*(1,9) = 1.102, *p* = 0.321, and interaction between them, *F*(1,9) = 0.157, *p* = 0.701.

##### The regulating effect of self-construal on the right superior temporal gyrus

In this part, we used the beta values of fMRI in ROI in four conditions, which was a 6-mm sphere with the center [60, -54, 21], as the dependent variables, humor and aggression as the in-group independent variables, and the self-construal as the between-group independent variable. The main effect of group, *F*(1,23) = 0.045, *p* = 0.835, was not significant. There was no significant main effect of aggression, *F*(1,23) = 0.018, *p* = 0.896, and interaction between self-construal and aggression, *F*(1,23) = 0.121, *p* = 0.731. The main effect of humor, *F*(1,23) = 10.631, *p* = 0.003, η^2^ = 0.316, power = 0.877, was significant, but the interaction between self-construal and humor, *F*(1,23) = 0.016, *p* = 0.899, was not significant. However, both the interaction between humor and aggression, *F*(1,23) = 12.493, *p* = 0.002, η^2^ = 0.352, power = 0.923, and the interaction among self-construal, humor, and aggression, *F*(1,23) = 5.371, *p* = 0.030, η^2^ = 0.189, power = 0.603, showed significance.

**FIGURE 9:**
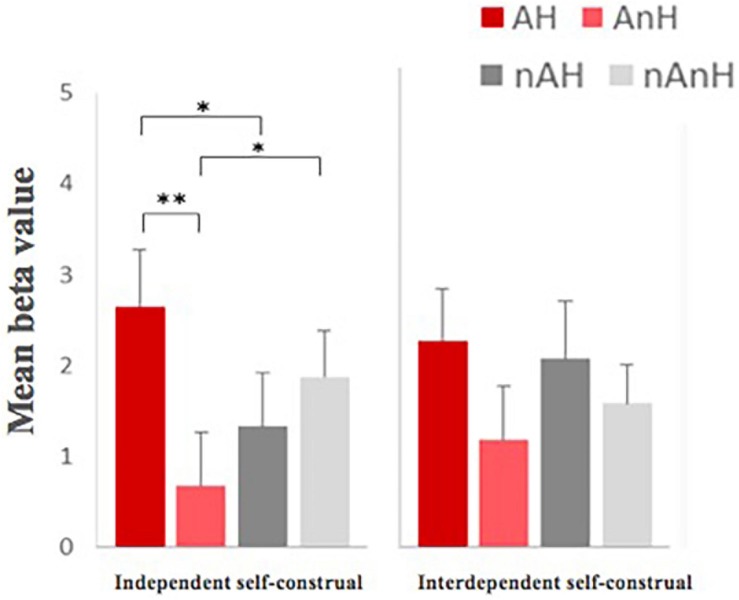
Different activation patterns in the right superior temporal gyrus [60 −54 21] between the independent self-constraul group and the interdependent self-construal group (^∗^ means *p* < 0.05, ^∗∗^ means *p* < 0.01, the 95% confidence interval).

The activation patterns of the brain region in the interdependent self-construal group and the independent self-construal group were significantly different under four conditions (Figure 9). For the independent self-construal group, the main effect of aggression, *F*(1,11) = 0.023, *p* = 0.883, was not significant. The main effect of humor, *F*(1,11) = 7.423, *p* = 0.020, η^2^ = 0.403, power = 0.699, was significant. The interaction between humor and aggression, *F*(1,11) = 13.419, *p* = 0.004, η^2^ = 0.550, power = 0.914, was significant, which manifested greater activation in AH than in AnH, *F*(1,11) = 13.922, *p* = 0.003, η^2^ = 0.559, power = 0.924, while no significant differences between nAH and nAnH, *F*(1,11) = 2.251, *p* = 0.162, and showed greater activation in AH than in nAH, *F*(1,11) = 6.614, *p* = 0.026, η^2^ = 0.376, power = 0.649, while less activation in AnH and nAnH, *F*(1,11) = 5.860, *p* = 0.034, η^2^ = 0.348, power = 0.597. For the interdependent self-construal group, there was no significant main effect of aggression, *F*(1,12) = 0.119, *p* = 0.736, main effect of humor, *F*(1,12) = 4.482, *p* = 0.056, and interaction between them, *F*(1,12) = 0.974, *p* = 0.343.

##### The regulating effect of self-construal on the right superior frontal gyrus

In the last part, we used the beta values of fMRI in ROI in four conditions, which was a 6-mm sphere with the center [15, 30, 48] as the dependent variables, humor and aggression as the in-group independent variables, and the self-construal as the between-group independent variable. The main effect of group, *F*(1,23) = 0.007, *p* = 0.933, was not significant. There was no significant main effect of aggression, *F*(1,23) = 0.709, *p* = 0.408, and interaction between self-construal and aggression, *F*(1,23) = 0.763, *p* = 0.391. There was no significant main effect of humor, *F*(1,23) = 3.559, *p* = 0.072, and interaction between self-construal and humor, *F*(1,23) = 0.384, *p* = 0.541. However, both the interaction effect between humor and aggression, *F*(1,23) = 17.051, *p* < 0.001, η^2^ = 0.426, power = 0.977, and the interaction effect among self-construal, humor, and aggression, *F*(1,23) = 4.878, *p* = 0.037, η^2^ = 0.175, power = 0.562, showed significance.

**FIGURE 10:**
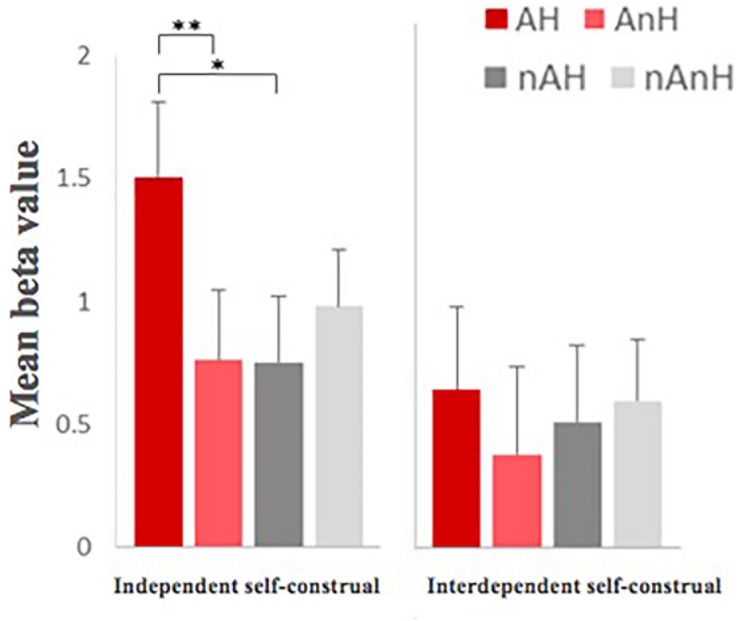
Different activation patterns in the right superior frontal gyrus [15, 30, 48] between the independent self-chonstrual and the interdependent self-construal group (^∗^ means *p* < 0.05, ^∗∗^ means *p* < 0.01, and ^∗∗∗^ means *p* < 0.001, the 95% confidence interval).

After further analysis of the interaction effect, the results showed that the activation patterns of the brain region between the interdependent self-construal group and the independent self-construal group were significantly different under four conditions (Figure 10). For the independent self-construal group, the main effect of aggression, *F*(1,11) = 1.163, *p* = 0.304, was not significant. The main effect of humor, *F*(1,11) = 3.098, *p* = 0.106, was not significant either. The interaction between them, *F*(1, 11) = 19.720, *p* = 0.001, η^2^ = 0.642, power = 0.981, was significant, which manifested greater activation in AH than in AnH, *F*(1,11) = 10.053, *p* = 0.009, η^2^ = 0.478, power = 0.823, while there was a significantly smaller greater activation in nAnH than in nAH, *F*(1,11) = 5.698, *p* = 0.036, η^2^ = 0.341, power = 0.586, and showed greater activation in AH than in nAH, *F*(1,11) = 7.907, *p* = 0.017, η^2^ = 0.418, power = 0.726, while no significant differences between AnH and nAnH, *F*(1,11) = 1.109, *p* = 0.315. For the interdependent self-construal group, there was no significant main effect of aggression, *F*(1,12) = 0.001, *p* = 0.980, main effect of humor, *F*(1,12) = 0.817, *p* = 0.384, and interaction between them, *F*(1,12) = 1.885, *p* = 0.195.

## Discussion

### Aggression Reduced the Degree of Funniness and Humor Reduced the Degree of Anger in Aggression Humor

The results suggested that aggression reduced the degree of funniness and humor reduced the degree of anger in aggression humor, which supported hypothesis 1.

For the degree of funniness, humor improved the degree of funniness of the materials significantly as we expected. In addition, we found that aggression would reduce the degree of funniness of the materials. In previous behavioral experiments, Epstein and Smith (1956) compared the degree of funniness of aggressive cartoons with non-aggressive cartoons and found that the former was higher than the latter. These two studies appeared inconsistent in the degree of funniness of aggression. It might be because Epstein didn’t consider whether their experimental materials were humorous or not. However, when we used a 2 × 2 design to compare the degree of funniness in AH, nAH, AnH, and nAnH, the present study showed that the aggression reduced the degree of funniness of the materials, instead of increasing it. Moreover, there was no definite direction of the materials in the previous study. It was possible that the point-to-self aggressive humor was more likely to trigger the anger of the recipients than the point-to-others aggressive humor. Humor and aggression had no significant interaction effect on the degree of funniness, indicating that they had an independent effect on the degree of funniness.

For the degree of anger, aggression improved the degree of anger of the materials in the present study, which was consistent with Anderson’s (2002) conclusion that aggressive attack could trigger anger. It suggested that higher aggressiveness apparently leads to decreased levels of pleasure (Willinger et al., 2017), which is inconsistent with our finding, probably because their materials were not point-to-self. It reminds us that the direction of humor can influence humor processing and therefore the future study of humor would pay attention on the direction of humor. In addition, humor would reduce the degree of anger of aggressive materials. It indicated that, as a high-level social communication skill, humor might have an alleviating effect on interpersonal conflicts. Consistent with the viewpoint of the superiority theory of humor (Wilkins and Eisenbraun, 2009), the results supported that when we expressed aggressive language inevitably, decorating our expression with humor could significantly reduce the anger that might be triggered, and humor showed its social adaptation function in this process. Besides, the significant interaction effect between humor and aggression indicated that the effect of humor on the degree of anger was associated with aggression, which would be further discussed by combining with fMRI results.

### Aggressive Humor Affected the Ratings of Funniness and Anger of Materials by Individual Differences of Self-Control and Self-Construal

Limited by the operation of MRI experiment, the sample size of the participants involved in this experiment was small. If the scores of self-control and self-construal of 25 participants were regarded as continuous variables directly, it might lead to an unstable description of the scores. Thus, we divided participants into high and low (high self-control/low self-control and independent self-construal/interdependent self-construal) groups according to their scores, and explored the correlates of personality variables with point-to-self aggressive humor processing by between-group comparison. For the degree of funniness of materials, the main effect of neither self-control group nor self-construal group was not significant. For the degree of anger of materials, although the main effects of two types of groups were not significant, the interaction among self-construal, aggression, and humor was significant, which showed that the independent self-construal group had greater difference of the anger degree between AH and AnH than the interdependent self-construal group. It indicated that the degree of anger of point-to-self aggressive humor materials decreased more in the independent self-construal group than in the interdependent self-construal group. In fact, a large number of studies in cultural psychology (Markus and Kitayama, 1991; Lutz, 2011) found that individuals with the character of independent self-construal paid more attention to individual interests and the expression of their own emotions, while individuals with the character of interdependent self-construal paid more attention to the collective interests and suppressed the expression of their own emotions. Based on this premise, the interdependent self-construal group would show lower anger degree of the aggressive materials in social communication, and thus the difference of anger degree between humor and non-humor was not significant. Correspondingly, for individuals in independent self-groups, their assessments of materials were more dependent on their emotional responses to materials. The humor significantly reduced their anger degree of aggressive materials.

### Neural Mechanism of the Aggressive Humor

For the neural mechanism of humor, the present study found that the left middle temporal gyrus/superior temporal gyrus, left triangle inferior frontal gyrus/orbital inferior frontal gyrus, right triangle inferior frontal gyrus/orbital inferior frontal gyrus, and left middle frontal gyrus showed stronger activation in the humor condition than in the non-humor condition, and the right occipital lobe was significantly inhibited. The activations of the left inferior frontal gyrus, middle temporal gyrus, and right inferior frontal gyrus were consistent with the results of previous studies: the left inferior frontal gyrus and middle temporal gyrus involved the cognitive process of humor, and the right inferior frontal gyrus involved the emotion process of humor (Bartolo et al., 2006; Chan et al., 2012; Amir et al., 2013). In order to distinguish different types of humor, the activated regions were further analyzed in AH and nAH conditions. It was found that the bilateral triangle inferior frontal gyrus/orbital inferior frontal gyrus and right angular gyrus/superior temporal gyrus/middle temporal gyrus were greater activated in AH than in AnH, while the nAH only activated the left middle temporal gyrus/inferior temporal gyrus and insular cap inferior frontal gyrus/triangle inferior frontal gyrus significantly compared to nAnH. As we can see, the left frontal lobe and the left temporal lobe associated with the cognitive component of humor were activated in both AH and nAH, but AH specifically activated the right frontal and the right temporal lobes. It suggested that humor was a high-level cognitive process, and different types of humor involved different brain regions. Nevertheless, humor processing still generally activated the left inferior frontal gyrus and the middle temporal gyrus. It inspired us when discussing the neural mechanism of humor processing; it was necessary to analyze the commonness and characteristics of different types of humor processing, so as to avoid over-generalization of the role of some brain regions involved in a certain type of humor processing.

The present study also found that the interaction between aggression and humor significantly activated three brain regions: (1) the right orbital inferior frontal gyrus, (2) the right dorsolateral superior frontal gyrus and medial superior frontal gyrus, and (3) the right superior temporal gyrus. The right orbital inferior frontal gyrus has been shown to be associated with the control of anger by a great deal of studies (Blair et al., 1999; Phan et al., 2002; Murphy et al., 2003). Since the point-to-self aggressive humor involved the complex emotional processing and played an important role in social adaptation, it was likely to involve the control of anger. The activation of the right orbital inferior frontal gyrus in the interaction indicated that this brain region played a role in anger control in the processing of point-to-self aggressive humor. The right dorsolateral superior frontal gyrus, medial superior frontal gyrus, and right temporal lobe were also activated, to which there were two possible explanations. Wittfoth et al. (2009) found that happy and angry existing in the meanwhile would activate these brain regions. The present research involved two conflict emotions, happy and angry. Hence, these regions might play an effect on cushioning the conflicts of emotions while the positive emotions and negative emotions presented at the same time, which was the first possible explanation. It supports the fact that the right frontal and temporal lobe were associated with self-cognition in plenty of MRI experiments, and the aggressive humor we used in present research was point-to-self. Thus, the second possible explanation we put forward was that these brain regions, which were specifically activated in the process of point-to-self aggressive humor, may also be the activation of self-cognitive neural network.

It was obvious that the three brain regions above were all located in the right brain regions. The result suggested that in the processing of point-to-self aggressive humor, there might be lateralization in brain regions, which played a key regulatory role in both aggression and humor. Based on this hypothesis, we further investigated the activated brain regions in four conditions. The right triangle inferior frontal gyrus, orbital inferior frontal gyrus, angular gyrus, superior temporal gyrus, middle temporal gyrus, and left triangle inferior frontal gyrus, orbital inferior frontal gyrus, middle temporal gyrus, and angular gyrus were activated in the AH condition, and the left medial temporal gyrus, inferior temporal gyrus, inferior insular gyrus, and inferior triangular gyrus were activated in the nAH condition. It was clear that the brain regions activated in the AH condition were relatively symmetrical, including multiple brain areas in the frontal lobe and temporal lobe, while the brain regions activated in the nAH condition showed obvious lateralization, which were mainly distributed in the left temporal lobe and the left frontal lobe. The results suggested that the left temporal lobe and the left frontal lobe might be associated with the point-to-self humor, and the right frontal and the right temporal lobes might be the specific key regions for processing point-to-self aggressive humor.

Although the neuropsychological research methods, which were mainly used by previous studies on the lateralization in humor processing (Gardner et al., 1975; Bihrle et al., 1986; Shammi and Stuss, 1999), could explore the role of different brain structures more directly in humor processing, its defects were obvious. On the one hand, the damaged regions and damaged degrees of different patients with brain injury were so different that there were many interfering factors in the study, and it was difficult to discuss more specific problems about humor processing through experimental operation. On the other hand, the neuropsychology method could only make such a rough localization of the brain region of humor processing that it was difficult to exclude the interference of the same region involving other cognitive functions. It might be because of the unavoidable interference and the lack of the variables controlling results of previous research on the lateralization in humor processing with inconsistent conclusions. However, the present study explored the differences in brain-activated patterns of the normal participants under different conditions by using fMRI event-related design, which could possibly avoid the differences caused by the different damaged regions and functions of the brain of different participants and reduce errors caused by the tasks involving other cognitive functions, and found that parts of the right frontal lobe and right temporal lobe were significantly activated in the processing of point-to-self aggressive humor.

According to the emotional valence hypothesis (Davidson, 1992), the left brain plays a dominant role in positive emotional processing, while the right brain plays a dominant role in negative emotional processing. The processing of point-to-self aggressive humor involves both the pleasure emotion triggered by conflict resolution and the anger emotion triggered by aggressive provocation. The present study showed the significant bilateral brain activation in the AH condition and the significant left-side brain activation in the nAH condition, which were consistent with the viewpoints of the emotional valence hypothesis (Davidson, 1992). Therefore, the lateralization of the point-to-self aggressive humor processing was probably because of the negative emotion. Additionally, since the materials in the study directed to the humor receiver themselves and the neural network of self-cognition involved the right frontal lobe and the right temporal lobe, the lateralization was probably related to the self-cognition (Calabrese et al., 1996; Fink et al., 1996; Craik et al., 1999; Keenan et al., 2000a, b).

The above results and discussion showed that hypothesis 2 was supported.

### Self-Control and Self-Construal Correlated With the Neural Mechanism of Aggressive Humor

Although the interaction between humor and aggression showed the activations in the right orbital inferior frontal gyrus, the right superior temporal gyrus, and the right superior frontal gyrus, the role of these brain regions in aggressive humor needed more evidence. Hence, we tested the correlation of self-control and self-construal with the neural mechanism of aggressive humor, and the results supported hypothesis 3.

For the right orbital inferior frontal gyrus, previous studies have shown that it played an important role in the control of anger in healthy people (Jollant et al., 2008). Tangney et al. (2004) proposed a scale that could measure the level of individual self-control and found that individuals with high self-control showed more rational in emotional responses. Therefore, comparing the differences between individuals with different levels of self-control in the right orbital inferior frontal gyrus can support the conclusion that this brain region played a role in anger control. As we expected, the high self-control group showed greater activation than the low self-control group in the right orbital inferior frontal gyrus, and the activation patterns of the two groups were significantly different. Different levels of self-control between individuals were associated with the right orbital inferior frontal gyrus. Considering that the self-control scale measures a wide range of self-control levels, including mental health, self-esteem, social skills, and emotions, the right orbitofrontal gyrus may also be associated with other self-control levels. There was no significantly different activation of the high self-control group in four conditions, while the low self-control group showed significant differences. Specifically, the low self-control group showed smaller activation in the AnH condition than in the AH and nAnH conditions. In other words, activation patterns in the right orbital inferior frontal gyrus of the low self-control group showed differences in the level of anger control on different types of materials, while the high self-control group showed no difference. These results also supported that the role of the right orbital inferior frontal gyrus on the neural processing of point-to-self aggressive humor correlated with anger control (Jollant et al., 2008).

Both the right superior temporal gyrus and the right superior frontal gyrus were important components of the neural network related to self-cognition, and self-construal was an indicator of the characterization of self-concept. Studies of cultural psychology suggested that individuals in the interdependent self-construal group paid more attention to collective interests, while individuals in the independent self-construal group paid more attention to individual interests. The impacts of self-construal involved cognition, emotion, and other aspects (Lutz, 2011). If the mechanism of the right superior temporal gyrus and the right superior frontal gyrus in the processing of point-to-self aggressive humor was related to self-cognition, we supposed there were different activation patterns between the interdependent self-construal group and the independent self-construal group in these two brain regions. As we expected, the right superior temporal gyrus and the right superior frontal gyrus showed similar activated patterns: there was no significantly different activation of the interdependent self-construal group between four conditions, while the independent self-construal group showed significant activation in the humor condition and significant interaction effect between humor and aggression. Specifically, the independent self-construal group showed greater activation in AH than in AnH, and no different activation between nAH and nAnH. It supported the fact that the role of the right superior temporal gyrus in the processing of point-to-self aggressive humor was correlated with the self-construal of individuals, and it also verified the relationship between this brain region and self-cognition.

### Implications and Innovations

First, the present study directly explored the neural processing mechanism of point-to-self aggressive humor, a common but special social phenomenon. Second, the study cleared humor and aggression and found that humor could significantly reduce the degree of anger caused by aggressive materials and verified the social adaptation value of humor, which could also explain the inconsistent conclusion in previous studies about “whether aggression makes people happy” and the lateralization of brain in humor processing. Third, the classification of humor was limited to the form of humor materials in previous studies about humor, and the present study concerned the different social attributes of humor and indicated that there were differences in the neural mechanism and rating results between different social attributes of humor, which was the first neural study supporting the model of humor style. Fourth, combining the self-control and self-construal, the study analyzed individual differences in processing point-to-self aggressive humor and made it support the key role of the brain regions in processing point-to-self aggressive humor.

### Limitations and Future Directions

Since the processing characteristics of point-to-self aggressive humor in the current study were obtained by comparing the two conditions, AH and nAH, which were point-to-self, we cannot determine “the effect of the aggressive humor on the processing mechanism.” The follow-up study could carry out the evaluation of humor and aggression materials and generate more horizontal continuous variables about them. To further explore the processing mechanism of point-to-self aggressive humor, it is beneficial for us to compare the changes in the processing of “aggression” and “humor,” which are continuous variables.

Further, in order to verify the mechanism of the right orbital inferior frontal gyrus and right superior frontal gyrus/superior temporal gyrus in the processing of point-to-self aggressive humor, the study combined the measurement results of the self-control and self-construal scale. As mentioned above, to avoid the interference of unstable score characterization caused by the sample size of the participants, we divided the participants into a high-score group and a low-score group, according to their scores of each personality, and compared the difference between groups. It is reasonable to make a conclusion that different groups showed different processing mechanisms of point-to-self aggressive humor but not extend to the effect of self-control and self-construal on the processing of point-to-self aggressive humor. However, the reliability of the self-construal scale was low, which may be due to the small number of participants. Subsequent studies may consider increasing the sample size and using self-control and self-construal level scores as continuous variables in the future.

Researchers found that when happy and angry appeared at the same time, they activated not only the regions associated with these emotions but also the regions associated with emotional conflict such as the right dorsolateral superior frontal gyrus, middle temporal gyrus, and superior temporal gyrus, which were different from the regions activated by processing cognitive conflict (Wittfoth et al., 2009). According to the characteristics of complex emotions that may be triggered by point-to-self aggressive humor, we speculated that the right superior temporal gyrus and the right superior frontal gyrus specifically activated by point-to-self aggressive humor in the present study may also be associated with the processing of emotional conflict. To further explore, future studies can investigate the relationship between the degree of anger and funny and the activation state of these brain regions based on a larger sample size.

## Conclusion

The present study examined the social attribute of humor and the processing mechanism of point-to-self aggressive humor. Humor can reduce the anger degree of the aggression people received, performing its function of social adaptation. The neural mechanism shows that the processing of point-to-self aggressive humor is associated with self-control and self-construal. These findings may provide a new perspective for studying the social attribute of humor and the processing of emotional conflict. We hope that these findings would be useful in future discussions on the theory of humor processing.

## Data Availability

The raw data supporting the conclusions of this manuscript will be made available by the authors, without undue reservation, to any qualified researcher.

## Ethics Statement

This study was approved by the Committee for Protecting Human and Animal Subjects at School of Psychological and Cognitive Sciences of Peking University, China. All subjects gave written informed consent in accordance with the Declaration of Helsinki.

## Author Contributions

All authors conceived the study, edited the manuscript, and approved the final version. XL and YC wrote the first draft of the manuscript. XL and LM contributed to the additional writing. YC, XL, and JG contributed to the data collection and analysis.

## Conflict of Interest Statement

The authors declare that the research was conducted in the absence of any commercial or financial relationships that could be construed as a potential conflict of interest.
