# Supplementary material for: Funny or Angry? Neural Correlates of Individual Differences in Aggressive Humor Processing
Source: Front Psychol. 2019 Aug 21;10:1849. doi: 10.3389/fpsyg.2019.01849 (PMC6712686; doi:10.3389/fpsyg.2019.01849)
Supplement: Supplementary file 1 [file Data_Sheet_1.PDF]

A Supplementary for "Funny or Angry? Neural Correlates of Individual Differences in Aggressive Humor Processing"

Here are English translations of the materials of four condition in our study.

AH condition

You are a teacher. One day in class, xiao Ming always talks.
Xiao Ming said to you very calmly:
"teacher, I am also very dissatisfied with you, but I never told your father."

Teachers said to you: "it is not allowed to wear other clothes unless uniforms in the school !"
But the teacher came up to you and said, "you can wear your own clothes."
Your face lit up and you asked, "why?"
The teacher said, "In order not to tarnish the image of the school, you'd better dress well."

The weather forecast said there would be a sun, but the rain kept falling for a week.
You say to your friend, "the weather is as changeable as your mood!"
Friend not to be outdone: "this weather forecast as unreliable as you!"

You're having a drama class. The teacher says to you: "classmate, your script in the gunshot too many."
You retort, "yes, but it's a military drama."
"But don't you think the sound of the gun will wake up the sleeping audience?" said the teacher.

You run a fruit store. "How expensive these apples are!" complained the customer.
You replied, "you get what you pay for. Look how red my apples are."
The customer retorted:
"Yes, the price is so high that even the apples are blushing for you!"

One of your friends loves writing. You said to him: "you have already made such a description in your works for the third time. Don't you know that the first one who compares women to flowers is a genius, and the third one is a mediocrity. 'is that famous?"
He retorted:
"Yes, you're right. You've used it for the seventh time."

When a friend owes you money, you ask him, "why have you been so slow in paying?"
"Don't worry," he said. "I'm waiting for two miracles. The first is the jackpot..."
You say: "you are crazy. How could such a thing happen? What was the second miracle?"
He replied, "and second, you must die."

You think you're the smartest person in the world and you brag about it. You say to an old man one day, "clever people are hard to find in this world." The old man said calmly:
"yes, because only the smart can find the smart, and the stupid, of course, is another matter."

You look very average, every time you look in the mirror to sigh. Once you saw yourself looking so bad, you looked in the mirror and cried.
A classmate came up to comfort you:
"You are like this, so we look at you every day, don't want to commit suicide? "

You take care of your friend's baby. The sleeping child began to cry again.

You decide to sing a lullaby.
No sooner had you started than your friend protested:
"Let the baby cry. He cries much better than you sing."

You say to a fired employee, "I hear you're going to spit on my grave after I die?"
The employee replied:
"Don't worry, I've changed my mind, I don't have the patience to wait in line!"

You say to a friend, "I'm really mad that my well-written paper was torn up by my three-year-old nephew."
The friend say:
"It seems that this three-year-old has read and correctly evaluated your paper!"

You ask a fortune-teller, "what day will my misfortune come?"
The fortune-teller replied, "in a festival."
You wonder.
The fortune-teller continued:
"For others, the misfortune of someone like you is a cause for celebration."

You say to the person you like, "I want to look at the moon and count the stars with you."
The other side say: "you this intelligence quotient, we can only see a star together, count a moon."

You finish your driver's license and ask the coach, "how did I do? Can my dad buy me a car?"
The coach says to you:
"Will one be enough?"

When you and your lover quarrel, you angrily say: "I was a complete fool that married you!"
The lover replies:
"Yes, but I was so infatuated with love that I didn't see it."

You blame your lover: "you never change."
The lover replied:
"If I had the courage, I would have divorced you."

After dinner, you and your mom watch TV news in the living room. When you see a piece of news, you angrily said: "this mother is too hateful, their own gambling money, lose broke, and finally sold their children! Mom, you wouldn't do that, would you?"
Your mom answered bluntly:
"Of course not. How can you mortgage worthless things?"

When you became an official, a poor friend came to congratulate you. You pretended not to know him,"who are you?
The friend was a little embarrassed at first, and then said calmly,
'they say you're blind and sick, so I came to see you. You don't even know your old friends."

You say to a friend, "I used to have your hair the way you do, but I thought it was ugly, so I changed it."
Your friend replied:
"I used to have a face like yours, but then I felt ugly, so I had to wear my hair like this."

You buy a fish from the street and say to your roommate, "you have to cook this fish. I will go to see a movie and then we will have dinner together."
When your roommate wants to go to the movies with you, you say, "I'll tell you about the movie after you cook dinner at home." Then you walked away.
You come home from the movie and ask for fish.
Your roommate said:
"I've eaten the fish. Now I'll tell you what it tastes like."

You solemnly said to your friend, "it was a mistake for you to refuse Xiao Tang. Now we cooperate."
The friend say:
"It is not surprised. When I refused Xiao Tang, he said he would do something extremely stupid when he was in pain."

The teacher is handing out math papers. You got a 0 and you took it.
The teacher said,
"Be careful! Take it in two hands, and don't break the egg."

After the shooting examination, the officer is not satisfied with your shooting results.
You say sadly, "this performance makes me want to shoot myself."
The chief say,
"You want to shoot yourself? That's not easy. You need more bullets."

You like singing. After singing a song at a karaoke bar, you return to your seat and excitedly say to your friend, "how did I do?"
The friend say:
"Well sung! But it's better not to sing."

AnH condition

You are a teacher. One day in class, Xiao Ming always talks.
Xiao Ming said to you very calmly:
"That is because you are too boring in class, I just talk to others."

The teacher said to you: ""it is not allowed to wear other clothes unless uniforms in the school !"
But the teacher comes to you and says, "especially you."
You ask curiously: "why?"
The teacher said,
"You're so ugly, don't waste your mind."

The weather forecast said there would be a sun, but the rain kept falling for a week.
You say to your friend, "the weather is as changeable as your mood!"
Friends not to be outdone:
"Your mood is more fickle than me, you have no qualify to say me."

You're having a drama class. The teacher says to you: "classmate, your script in the gunshot too many."
You retort, "yes, but it's a military drama."
The teacher said,

"Your script is boring, and all that shooting just makes the audience more upset."

You run a fruit store. "How expensive these apples are!" complained the customer.
You replied, "you get what you pay for. Look how red my apples are."
The customer retorted:
"Look at your red apples. Many of them have worms. I don't have the nerve to sell so expensive even if the quality is not good."

One of your friends loves writing. You said to him: "you have already made such a description in your works for the third time. Don't you know that the first one who compares women to flowers is a genius, and the third one is a mediocrity. 'is that famous?"
He retorted:
"That is you do not appreciate, do not comment without the ability to comment."

When a friend owes you money, you ask him, "why have you been so slow in paying?"
He said, "don't worry, I'm waiting for a miracle. I hope I win the lottery..."
You say: "you are crazy. How could such a thing happen?"
He replied:
"You are too mean to haggle over a little money."

You think you're the smartest person in the world and you brag about it. You say to an old man one day, "clever people are hard to find in this world."
The old man said calmly:
"From what I know of you, you are not clever at all, but a fool."

You look very average, every time you look in the mirror to sigh. Once you saw yourself looking so bad, you looked in the mirror and cried.
A classmate came up to comfort you:
"You're ugly enough, and crying is even worse."

You take care of your friend's baby. The sleeping child begin to cry again. You decide to sing a lullaby.
No sooner had you started than your friend protested:
"You're a terrible singer. Stop singing."

You say to a fired employee, "I hear you're going to spit on my grave after I die?"
The employee replied:
"Of course I'm going to spit on a selfish of the person like you who squeezes people's labor."

You say to a friend, "I'm really mad that my well-written paper was torn up by my three-year-old nephew."
The friend say:
"Your paper is so badly written that it would be a pity if it was torn up."

You ask a fortune-teller, "what day will my misfortune come?"
The fortune-teller replied, "in a festival."
You wonder.
The fortune-teller continued:
"It's because you're evil that something bad will happen to you at Christmas tomorrow."

You say to the person you like, "I want to look at the moon and count the stars with you."
The person said,

"Because you're so stupid, I'm not interested in you."

You finish your driver's license and ask the coach, "how did I do? Can my dad buy me a car?"
The coach says to you:
"You are so bad that buying a car is a threat to passers-by."

When you and your lover quarrel, you angrily say: "I was a complete fool that married you!"
The lover replies:
"The stupidest thing I ever did was marry you."

You blame your lover: "you never change."
The lover replied:
"You are incapable, ill-tempered, and have never changed."

After dinner, you and mom watch TV news in the living room.
When you see a piece of news,you angrily said: " this mother is too hateful, their own gambling money, lose broke, and finally sold their children! Mom, you wouldn't do that, would you?"
Your mom answered bluntly:
"You are so lazy and make me angry every day. I really want to sell you."

When you became an official, a poor friend came to congratulate you. You pretended not to know him. "who are you?”
The friend was a little embarrassed at first, and then said calmly,
"Now that you're in office, you're so arrogant and complacent that you don't even recognize your old friends."

You say to a friend, "I used to have your hair the way you do, but I thought it was ugly, so I changed it."
Your friend replied:
"I think your hair is worse than your old one, so change it."

You buy a fish from the street and say to your roommate, "you have to cook this fish. I will go to see a movie and then we will have dinner together."
When your roommate wants to go to the movies with you, you say, "I'll tell you about the movie after you cook dinner at home." Then you walked away.
You come home from the movie and ask for fish.
Your roommate said:
"Why should I cook your fish when you're so selfish?"

You solemnly said to your friend, "it was a mistake for you to refuse Xiao Tang. Now we cooperate."
The friend say:
"Xiao Tang has no ability and is not a good person. Working with him shows that you have a bad eye."

The teacher is handing out math papers. You got a 0 and you took it.
The teacher said,
"So bad at exams and so bad at tempers."

After the shooting examination, the officer is not satisfied with your shooting results.

You say sadly, "this performance makes me want to shoot myself."
The chief said.
"Your grades are so bad that killing yourself won't be a loss."

You like singing. After singing a song at a karaoke bar, you return to your seat and excitedly say to your friend, "how did I do?"
The friend say:
"It's terrible. You'd better stop singing."

nAH condition

You're good at running. In a track meet, you finished last because of an injury.
You ask your friend, "will I lose face when I finish last?"
He said:
"On the contrary, don't you see them all being chased by you? You're sure to catch up with them next time."

You walk into a supermarket and ask for half a cabbage.
The assistant tells you that the supermarket only sells whole cabbages.
But you insist on only half a cabbage.
The assistant said he must consult the manager. The assistant walked into the manager's office and said, "manager, there is a fool outside who wants to buy half a cabbage."
As he speaks, the assistant turns around and finds you standing behind him.
He immediately continued:
"Fortunately, the respected guest asked for the other half."

You tell your friend, "you have no idea how miserable my life is! When my wife gets angry, she yells at me, at the kids, even at our puppy, and none of us dares utter a word. What shall I do when I am angry?"
Your friend replies:
"You can shout at the walls, the Windows and the doors, none of them will speak."

Your friend: "hear you very afraid teacher?"
You: "who said that? I'm only afraid sometimes."
Friend:
"Sometimes? It means you afraid when you stay with the teacher!"

You're a thoracic surgeon. Your car broke down and you take it to the repair shop. There's something wrong with the engine. The mechanic deftly dismantled and reinstalled the engine. You ask the mechanic, "the engine is the heart of the car, we all repair the heart, but why is the income gap so big?"
The repairman thought about it and said,
"Because you did it without flameout."

You and your friends see a group of young people in the street wearing strange clothes and with their hair dyed red and green.
You sighed a breath to say: "now of the child, whom the heart is pale, can only use the appearance of fashion to cover up the essence of their vulgar."
The friend smiled and said:
"Yes, the elegant person like you is different, you can only desperately with the appearance of your vulgar to cover up the essence of fashion."

On your birthday, you melancholy soliloquize: "turn an eye to more than 20 years old!"
A friend comfort you,
"One tenth of life is gone!"

Your boss has a hot temper and always beat or scold you.
One day you can't stand it. You ask a friend, "how can I stand such a boss?"
Your friend replied:
"If you have a good-natured boss, you will be happy; If you have a bad-tempered boss, you will become a successful person."

You have bought keyboard, and going to the supermarket to buy two big iron bowls.
A friend of yours happens to see you and says,
"You must have broken the keyboard on your knees yesterday and hit the bowl you put on your head accidentally."

Your classmate ask you: "last night your bedroom have very big voice, exactly what matter?"
You say, "no big deal, my roommate is a little upset and threw my coat down the stairs."
Your classmate:
"It's so loud that you must have it wrapped up in your coat."

You complain to your friends: "my wife takes care of all the money in my family, and I have no status!"
Your friend say:
"Your spouse takes care of the money. You take care of your spouse. You have both of them!"

In class, the professor asks you to give a presentation. You lost your footing on the platform and nearly fell.
When the professor saw you steady yourself, he said to you:
"How hard it is to go up a height! Life is like this, so is learning!"

You haven't talked for days after you quarreled with your roommate. Today, the roommate returned to the bedroom and see you are still in the sulk. He quietly turn over things, drawer, wardrobe all toss out clothes, take them all over the floor. "What are you looking for?" you ask.
Your roommate replied:
“thank god! I'm looking for your voice!"

You're bumping into someone head-on when you walk with your head down. You apologize and say, "I'm sorry, I must be blind."
The other smiled and said:
"You have good eyes, didn't you just hit me?"

The teacher handed out the test paper, and asked everyone to get parents to sign. The next day, the teacher asked you, "didn't you show the test paper to your parents? Why didn't they sign it?"
You hold out your whip-scarred hands.
The teacher said,
"It is the signed here."

You went out and bought a pair of expensive leather shoes. Later, you complained to your colleagues: "I'm too impulsive. A pair of shoes cost me half of my monthly salary!"
Your colleagues said,
"Thanks to our evolution to walk upright, four feet would have cost you a month's salary."

You have a hemorrhoid surgery and your colleagues are meeting to visit you in the hospital. Peoples stand in the ward, who are too embarrassed to ask your condition. Xiao Li, the driver of the company, let out a dry cough and asked you seriously:
"I heard there was something wrong with your chassis. Is it all right now?"

You are an amateur writer, chatting with a friend one day.
You say: "we literati have a good appetite, we eat anything in our pen: bear hardships, bear weight (strenuous) , have vinegar (jealous) , have lawsuit (accused) , have northwest wind (poor) , chew character (chop logic) ... what not to eat?"
Your friend replied:
"You will not eat soft (don't accept bribes) , hard(not afraid of threat), or short-sighted food (don't suffered losses) ."

You did well in your major, but it took too long to write your paper.
One day you laughed to yourself and said, "god made a world in seven days. Why does it take me so long to write a paper?"
Your classmate say:
"You look at god's terrible world, and you look at this great paper you've written..."

You sit in a cafe with an old friend.
Your friend say: "what was your dream as a child?"
You say: "be a policeman. It seems that my dream has been shattered."
Friend:
"You're halfway there. At least you're an 'man' now."

Qiang is a staff in your shop. One morning, he went to hang a sign, accidentally, the sign fell in half.
You get angry and say, "why are you so careless? Leave again like this!"
Qiang said calmly:
"You don't be angry, sign change two half to say you want to open a branch, this is very good omen!"

You are a good jumper, but you didn't get any prize in this sports meet.
When you see the sports committee,you chagrin and say: "I was a high jump champion! Are you disappointed in me?"
He thought for a moment, said in smile:
"Every class wants to be proud of being the class champion. If you always win, what about the other classes?"

You have a little garden behind your house. You grow vegetables in the garden, but the ducks of the neighborhood always come and steal the vegetables.
A few days before the Spring Festival, your neighbor brought you a roast duck. You show gratitude to your neighbors.
The neighbor said:
"You're welcome, in fact this duck is your vegetables change come."

You stay up late reading in the ATM cubicle.

A classmate found out and asked: "what are you doing here?"
You say, "read a book."
"Why are you reading here?" he asked.
Before you answer,he said to himself:
"I've learned that when you can't read it, just insert your card into the ATM to check your balance,you'll be motivated to keep reading."

You're a student assistant at the weather bureau. One day,the meteorologist tells you to write down the weather forecast for Sunday : It will be cloudy in the morning and rainy in the afternoon.
"I'm going to the amusement park on Sunday afternoon," you say with a sigh.
The meteorologist said sympathetically:
"Cut out the rain, then."

nAnH condition

You're good at running. In a track meet, you finished last because of an injury.
You ask your friend, "will I lose face when I finish last?"
He said:
"it was understandable that you came in with injury and your opponents played well this time."

You walk into a supermarket and ask for half a cabbage.
The assistant tells you that the supermarket only sells whole cabbages.
But you insist on only half a cabbage.
The assistant said he must consult the manager. The shopping assistant walked into the manager's office and said,
"Manager, there's a fool out there who wants to buy half a cabbage."
As he speaks, the assistant turns around and finds you standing behind him.
He immediately continued:
"Sorry, I am wrong, this customer wants half a cabbage."

You tell your friend, "you have no idea how miserable my life is! When my wife gets angry, she yells at me, at the kids, even at our puppy, and none of us dares utter a word. What shall I do when I am angry?"
Your friend replies:
"Then you have to hold it in your heart or do some vigorous exercise to relieve the mood."

Your friend say: "hear you very afraid teacher?"
You say: "who said that? I'm only afraid sometimes."
Friend:
"That must be when the teacher is angry!"

You're a thoracic surgeon. Your car broke down and you take it to the repair shop. There's something wrong with the engine. The mechanic deftly dismantled and reinstalled the engine.
You ask the mechanic, "the engine is the heart of the car, we all repair the heart, but why is the income gap so big?"
The repairman thought about it and said,
"Because your patient's life is at stake."

You and your friends see a group of young people in the street wearing strange clothes and with their hair dyed red and green.
You sighed a breath to say: "now of the child, the heart is pale, can only use the appearance of fashion to cover up the essence of their vulgar."
The friend smiled and said:
"The real fashion depend on the internal cultivation, cultivation improved, the external temperament will be shown."

On your birthday, you melancholy soliloquize: "turn an eye to more than 20 years old!"
A friend comfort you,
"So you should cherish the time later!"

Your boss has a hot temper and always beat or scold you.
One day you can't stand it. You ask a friend, "how can I stand such a boss?"
His friend replied:
"Learning to endure is a necessary part of life, so we have to endure the unchangeable facts of our life."

You have bought keyboard, and going to the supermarket to buy two big iron bowls.
A friend of yours happens to see you and says,
"You must have broken the bowl accidentally, so come to the supermarket to buy it."

Your classmate ask you: "last night your bedroom have very big voice, exactly what matter?"
You say, "no big deal, my roommate is a little upset and threw my coat down the stairs."
Your classmate:
"It was so loud that your roommate threw at least one tin down."

You complain to your friends: "my wife takes care of all the money in our family, and I have no status!"
The friend say:
"It's normal if your partner is better at managing money than you are,. It doesn't leave you without status."

In class, the professor asks you to give a presentation. You lost your footing on the platform and nearly fell.
When the professor saw you steady himself, he said to you:
"This step is too high. Be careful when you go to the podium."

You haven't talked for days after you quarreled with your roommate. Today, the roommate returned to the bedroom and see you are still in the sulk. He quietly turn over things, drawer, wardrobe all toss out clothes, take them all over the floor. "What are you looking for?" you ask.
Your roommate replied:
"I can't find my watch. I'm looking for it."

You're walking with your head down when you bump into someone head-on. You apologize and say, "I'm sorry, I must be blind."
The other smiled and said:
"You just don't walk carefully, that's ok."

The teacher handed out the test paper, and asked everyone to get parents to sign. The next day, the teacher asked you, "didn't you show the test paper to your parents? Why didn't they sign it?"
You hold out your whip-scarred hands.
The teacher said,
"They won't sign it, they beat you."

You went out and bought a pair of expensive leather shoes. Later, you complained to your colleagues: "I'm too impulsive. A pair of shoes cost me half my monthly salary!"
Your colleagues said,
"Thank god you only bought one pair. If you bought two pairs, it would be a month's salary."

You have a hemorrhoid surgery and your colleagues are meeting to visit you in the hospital. Peoples stand in the ward, who are too embarrassed to ask your condition. Xiao Li, the driver of the company, let out a dry cough and asked you seriously:
"I hear you haven't been feeling well lately. Are you better now?"

You are an amateur writer, chatting with a friend one day.
You say: "we literati have a good appetite, we eat anything in our pen: bear hardships, bear weight (strenuous) , have vinegar (jealous) , have lawsuit (accused) , have northwest wind (poor) , chew character (chop logic) ... what not to eat?"
Your friend replied:
"You're right. You have a good appetite."

You did well in your major, but it took too long to write your paper.
One day you laughed to yourself and said, "god made a world in seven days. Why does it take me so long to write a paper?"
Classmate say:
"Please don't complain. It's normal that you need to spend more time to ensure the quality of your paper."

You are sitting in a cafe with an old friend.
Your friend say: "what was your dream as a child?"
You say: "be a policeman. It seems that my dream has been shattered."
Friend:
"I just heard that you have passed the examination and may soon be able to work for the police."

Qiang is a staff in your shop. One morning, he went to hang a sign, accidentally, the sign fell in half.
You get angry and say, "why are you so careless? Leave again like this!"
Qiang said calmly:
"Boss, it's time to changed your sign. The sign is too weak."

You are a good jumper, but you didn't get any prize in this sports meet.
You see the sports committee, chagrin and say: "I was a high jump champion! Are you disappointed in me?"
He thought for a moment, smiled, and said:
"You forgot? You were sick for a week before the race and you weren't feeling well during the race so it must have affected your results. Come on next time!"

You have a little garden behind your house. You grow vegetables in the garden, but the ducks of the neighborhood always come and steal the vegetables.
A few days before the Spring Festival, your neighbor brought you a roast duck. You show gratitude to your neighbors.
The neighbor said:
"You're welcome, my duck always eat your vegetables, this is the compensate to you."

You stay up late reading in the ATM cubicle.
A classmate found out and asked: "what are you doing here?"
You say, "read a book."
"Why are you reading here?" he asked.
Before you answer,he said to himself:
"I see. Our school dormitory is out of power tonight, the study room is closed, and we have a final exam tomorrow, so you have to watch it here."

You're a student assistant at the weather bureau. One day,the meteorologist tells you to write down the weather forecast for Sunday : It will be cloudy in the morning and rainy in the afternoon.
"I'm going to the amusement park on Sunday afternoon," you say with a sigh.
The meteorologist said sympathetically:
"You have to change the date to the playground."

Here are the original materials of four condition in our study.

攻击幽默条件

你是一个老师，一天上课时，小明老是讲话，你就生气地说：“小明，你要是再不老实，我就告诉你爸爸。”
小明十分冷静地对你说：
“老师，我对你也很不满意，可是我从来没有告诉过你爸爸。”

老师对你们说：“在学校不允许穿校服以外的衣服！”
但老师走到你身旁说：“你可以穿自己的衣服。”
你面露喜色地问：“为什么呀？”
老师说：“为了不让学校形象受损，你还是穿得好看一点吧。”

天气预报说会出太阳，可是雨却淅淅沥沥下了一周不见停。
你对朋友说：“这天气就跟你的心情一样善变！”
朋友不甘示弱：“这天气预报就跟你一样不靠谱！”

你正在上一门戏剧方面的课。老师对你说：“同学，你的剧本里枪声太多了。”
你反驳说：“是的，可它是一部军事题材的戏剧。”
老师说：“可是，你不觉得枪声会把睡着的观众吵醒吗？”

你经营一家水果店。顾客抱怨道：“这些苹果真贵呀！”

你回答道：“一分钱一分货，你看我苹果多红呀。”
顾客反驳说：
“是啊，要价这么高，连苹果都替你们脸红了！”

你的一个朋友酷爱写作。你对他说：“你已经第三次在作品里做这样的描述了，难道你不知道‘第一个把女人比喻为花的是天才，第三个是庸才。’这句名言吗？”
他反驳道：
“是的，你说的对，你已经第七次使用这句话了。”

一个朋友欠了你钱，你问他：“你为何迟迟不还钱？”
他说：“你不要着急，我在等待两种奇迹的发生。第一是中大奖……”
你说：“你是异想天开，这样的事怎么可能发生呢？第二个奇迹是什么？”
他回答：“第二，你总得离开人世吧！”

你认为自己是世上最聪明的人并常常向人夸耀。有一天你对一位老人说：“这个世界上聪明人真是难找呀，这些年我去了很多地方，一个聪明人都没见过。”老人不动声色地说：
“是啊，因为只有聪明人才能发现聪明人，蠢货当然就另当别论了。”

你长得很一般，每当你照镜时都要感叹一下。有一次你看见自己长得很不乐观，对着镜子哭了出来。
一个同学走过来，安慰你：
“你都这样，那我们每天看着你，岂不是想去自杀了？！”

你帮朋友照顾宝宝。睡着了的孩子又哭了起来。
你决定唱一段催眠曲。
刚开了个头，你朋友就抗议了：
“还是让孩子哭吧。他哭得比你唱得好听多了。”

你对被开除的员工说：“听说，你要在我死后到坟场上对我的坟墓吐口水？”
员工回答道：
“放心吧，我已经改变了主意，我才没有排队的耐心呢！”

你对朋友说：“我真气坏了，好好写成的论文，被我三岁的侄子撕破了。”
朋友说：
“看来这个三岁的孩子已经识字并对你的论文做出正确的评价了！”

你问算命的人：“我会在哪一天遭遇不幸呢？”
算命的人回答说：“在一个节日里。”

你感到奇怪。算命的人继续说：
“对于别人来说，你这样的人遭遇不幸，就是一件值得庆祝的事情。”

你对你喜欢的人说：“我要和你一起看月亮，数星星。”
对方说：“就你这智商，我们只能一起看星星，数月亮。”

你考完驾照后问教练：“我表现如何?我爸可以买一部车给我了吧?”
教练回答你说：
“一部够吗？”

你和爱人在吵架时，生气说：“我和你结婚时，完全是一个傻瓜！”
爱人回答道：
“是的，只怪我当时被爱冲昏了头脑，没有发现。”

你埋怨爱人：“你总是知错不改。”
爱人回答说：
“我若有这种勇气，早就和你离婚了。”

晚饭后，你和妈妈在客厅看电视新闻。当你看到一则新闻时，气愤地说：“你看，这个妈妈太可恶了，自己去赌钱，输得倾家荡产，最后还把自己的孩子卖掉了！妈妈，你该不会也这么做吧？”
妈妈直截了当地回答：
“当然不会，不值钱的东西，怎么能拿去抵押呢？”

你当了官，一个穷朋友前来祝贺，你装作不认识：“你是谁呀，来这干嘛？”
朋友开始有点尴尬，后来镇定地说：
“人家都说你的眼睛得病瞎了，所以我来看看你。你果然是连老朋友都不认得了。”

你对朋友说：“过去我也留有你这样的发型，后来觉得挺难看的，就换掉了。”
朋友回答说：
“过去我也长着一张你这样的脸，后来觉得难看，就只好留这样的发型了。”

你从街上买回来一条鱼，对室友说：“你现在烧这条鱼，我去看电影，看完回来咱们一起吃晚饭。”
室友要和你一起去看电影，你说：“你在家里做饭，我看完后给你讲一讲电影的内容。”说罢扬长而去。
你看完电影回到家嚷着要吃鱼。
室友说：
“鱼我已经吃了，现在我给你讲一讲鱼的味道。”

你郑重地对朋友说："你拒绝小汤是一个错误，现在我们合作了。"
朋友说：
"我一点也不奇怪，当我拒绝小汤时，他就说，他在痛苦的时候会做一些极其愚蠢的事情。"

老师在发数学试卷。你考了 0 分，没好气地一把接过。
老师说：
"小心！两只手拿，别把蛋打破了。"

射击考核以后，长官对你的射击成绩很不满意。
你悲伤地说："这样的成绩都让我想开枪自杀。"
长官说：
"你想开枪自杀？那可不太容易。你要多带子弹才行。"

你是歌霸，在卡拉 OK 厅唱罢一曲，回到座位兴奋地对朋友说："我唱得如何？"
朋友说：
"唱得很好！不过，不唱更好。"

攻击非幽默条件

你是一个老师，一天上课时，小明老是讲话，你就生气地说："小明，你要是再不老实,我就告诉你爸爸。"
小明十分冷静地对你说：
"那是因为你上课太无聊了，我才和别人讲话的。"

老师对你们说："在学校不允许穿校服以外的衣服！"
但老师走到你身旁说："尤其是你。"
你奇怪地问："为什么呀？"
老师说：
"你长得那么丑，穿什么都一样，就不要浪费心思了。"

天气预报说会出太阳，可是雨却淅淅沥沥下了一周不见停。
你对朋友说："这天气就跟你的心情一样善变！"
朋友不甘示弱：
"你的心情比我更加善变，没有资格说我。"

你正在上一门戏剧方面的课。老师对你说："同学，你的剧本里枪声太多了。"
你反驳说："是的，可它是一部军事题材的戏剧。"
老师说：
"你的剧本内容枯燥，那么多枪声只会让观众更加烦躁。"

你经营一家水果店。顾客抱怨道：“这些苹果真贵呀！”
你回答道：“一分钱一分货，你看我苹果多红呀。”
顾客反驳说：
“你看你的红苹果，好多都有虫眼。质量不好还好意思卖那么贵。”

你的一个朋友酷爱写作。你对他说：“你已经第三次在作品里做这样的描述了，难道你不知道‘第一个把女人比喻为花的是天才，第三个是庸才。’这句名言吗？”
他反驳道：
“那是你不懂欣赏，没有评论的能力就不要评论。”

一个朋友欠了你钱，你问他：“你为何迟迟不还钱？”
他说：“你不要着急，我在等待奇迹的发生。希望我可以中大奖……”
你说：“你是异想天开，这样的事怎么可能发生呢？”
他回答：
“你太小气了，那么一点钱还要斤斤计较。”

你认为自己是世上最聪明的人并常常向人夸耀。有一天你对一位老人说：“这个世界上聪明人真是难找呀，这些年我去了很多地方，一个聪明人都没见过。”
老人不动声色地说：
“以我对你的了解，你一点都不聪明，而是一个蠢货。”

你长得很一般，每当你照镜时都要感叹一下。有一次你看见自己长得很不乐观，对着镜子哭了出来。
一个同学走过来，安慰你：
“你已经够难看了，哭了更难看。”

你帮朋友照顾宝宝。睡着了的孩子又哭了起来。你决定唱一段催眠曲。
刚开了个头，你朋友就抗议了：
“你唱的太难听了，不要再唱了。”

你对被开除的员工说：“听说，你要在我死后到坟场上对我的坟墓吐口水？”
员工回答道：
“像你这样压榨员工劳动力的自私自利的人，我当然要去吐口水。”

你对朋友说：“我真气坏了，好好写成的论文，被我三岁的侄子撕破了。”
朋友说：
“你的论文写得太差了，被撕了也不可惜。”

你问算命的人：“我会在哪一天遭遇不幸呢？”
算命的人回答说：“在一个节日里。”
你感到奇怪。算命的人继续说：
“因为你心肠歹毒，所以明天圣诞节就会遭遇不幸。”

你对你喜欢的人说：“我要和你一起看月亮，数星星。”
对方说：
“因为你太笨了，我对你不感兴趣。”

你考完驾照后问教练：“我表现如何?我爸可以买一部车给我了吧?”
教练回答你说：
“你技术太差了，买车只会对路人造成威胁。”

你和爱人在吵架时，生气说：“我和你结婚时，完全是一个傻瓜！”
爱人回答道：
“我和你结婚，才是我做的最傻的事情。”

你埋怨爱人：“你总是知错不改。”
爱人回答说：
“你自己没能力，脾气差，也从来没改过。”

晚饭后，你和妈妈在客厅看电视新闻。
当你看到一则新闻时，气愤地说：“你看，这个妈妈太可恶了，自己去赌钱，输得倾家荡产，最后还把自己的孩子卖掉了！妈妈，你该不会也这么做吧？”
妈妈直截了当地回答：
“你那么懒惰，每天惹我生气，我真的很想把你卖掉。”

你当了官，一个穷朋友前来祝贺，你装作不认识：“你是谁呀，来这干嘛？”
朋友开始有点尴尬，后来镇定地说：
“你现在当官了，就目中无人，得意忘形，连老朋友都不认了。”

你对朋友说：“过去我也留有你这样的发型，后来觉得挺难看的，就换掉了。”
朋友回答说：
“我觉得你现在的发型比原来的更加难看，还是再换一个吧。”

你从街上买回来一条鱼，对室友说：“你现在烧这条鱼，我去看电影，看完回来咱们一起吃晚饭。”
室友要和你一起去看电影，你说：“你在家里做饭，我看完后给你讲一讲电影的内容。”说罢扬长而去。
你看完电影回到家嚷着要吃鱼。
室友说：
“你那么自私自利，我为什么要帮你做鱼。”

你郑重地对朋友说：“你拒绝小汤是一个错误，现在我们合作了。”
朋友说：
“小汤既没有能力，人品也不怎么好，真可以说是一无是处。跟他合作说明你的眼光很差。”

老师在发数学试卷。你考了 0 分，没好气地一把接过。
老师说：
“考试成绩那么差，脾气还那么差。”

射击考核以后，长官对你的射击成绩很不满意。
你悲伤地说：“这样的成绩都让我想开枪自杀。”
长官说：
“你的成绩那么差，自杀了也不会是一种损失的。”

你是歌霸，在卡拉 OK 厅唱罢一曲，回到座位兴奋地对朋友说：“我唱得如何？”
朋友说：
“唱得太难听了，你以后还是不要唱了。”

非攻击幽默条件

你很擅长跑步。在一次田径比赛中，你因伤跑了最后一名。
你问你的朋友：“我跑了最后一名会不会很没有面子？”
他说道：
“恰恰相反，你没有看到他们几个人都被你追的直跑吗？下次你肯定能追上他们。”

你走进一家超市，要求购买半棵白菜。
导购员告诉你，超市只出售整棵白菜。
但你坚持只要半棵白菜。
导购员表示，必须请示经理。导购员走进经理办公室说：“经理，外面有个傻子要买半棵白菜。”
说话时，导购员回过头，发现你站在他身后，
他立刻接着说：
“幸好，这位贵客说要买另一半。”

你向你的朋友诉苦：“你不知道我现在的生活过得有多憋屈！当我的妻子发怒时，她会对着我、孩子，甚至对着我们的小狗大喊大叫，而我们谁都不敢吭一声。当我发怒的时候，怎么办呢？”
你的朋友答道：
“你可以冲着墙壁、窗户和门大喊大叫，他们谁也不敢吭声。”

朋友：“听说你很怕老师？”
你：“谁说的？我只是某些时候才怕。”
朋友：
“某些时候？你指的是和老师在一起的时候吧！”

你是一名胸外科医生。你的车坏了，送到修理部检查，是引擎出了毛病。修理工熟练地把引擎拆下来又装上。
你问修理工：“引擎就是汽车的心脏，我们都是修理心脏的，可是收入为什么差距那么大？”

修理工想了想说：
“因为你们是在不熄火的情况下进行修理的。”

你和朋友在大街上看到一群年轻人穿着奇装异服，染着红红绿绿的头发。
你叹了一口气说：“现在的小孩子，内心苍白，只能用外表的时髦来掩饰本质上的土。”
朋友笑着说：
“是啊，像你这种高雅的人就不同了，只能拼命用外表的土来掩饰本质的时髦。”

又过了一个生日，你惆怅自语：“唉，转眼就二十多岁了！”
朋友要安慰你，说：
“人生的十分之一就这么过去了！”

你的老板脾气火爆，对你非打即骂。
你有一天实在忍受不了，就问朋友：“这样的老板我怎么能够忍受得了？”
朋友回答说：
“如果你有个脾气好的老板，你会很幸福；如果你有个脾气不好的老板，你会成为一个事业有成的人。”

你去买键盘，买完后你又到超市买了两个大铁碗。
你一个朋友正好看到你，就说：
“你一定是昨天把键盘跪断了，一不小心顶在头上的碗也打了。”

同学问你：“昨夜你寝室有很大的声音，到底什么事？”
你说：“没什么大事，我室友有点不高兴，把我的大衣从楼上扔下去。”
同学：
“声音那么大，你一定是在大衣里包着。”

你和朋友抱怨：“我们家的钱都是我爱人管的，我也太没有地位了！”
朋友说：
“你爱人管钱，你来管你爱人。那么这样一来，你就人财两得了！”

上课时，教授请你上台做展示。你在上讲台时没有站稳，差点跌倒。
教授见你稳住身体，对你说：
“你看，上升一个高度多么不容易！生活是这样，学习也是这样！”

你和你的室友吵架后，几天没有说话。这天，室友回到寝室，见你仍在怄气，便不声不响地翻起东西来，把抽屉、衣柜里的衣物全部折腾出来，弄得乱七八糟，满地都是。你实在忍不住了，问道：“你到底在找什么？”
室友回答说：
“谢天谢地！我在找你的声音！”

你正在低头赶路，迎面撞上一个人，就道歉说：“对不起对不起，我眼睛瞎了。”
对方笑了笑说：

“你眼睛很好啊，刚刚不是把我撞个正着吗？”

老师把试卷发下来，并要求大家给父母看后请父母在上面签字，再交回来。第二天，老师问你：“你没有把试卷给父母看吗，为什么没有签字？”
你伸出满是鞭痕的手。
老师说：
“原来字就签在这儿。”

你上街买了一双挺贵的皮鞋。后来跟同事抱怨说：“都怪我太冲动，一双鞋就花了我半个月工资！”
同事说：
“多亏人类进化到直立行走，否则，就要花掉你一个月工资了。”

你做了一个痔疮手术，同事们约着一起去医院探望。男男女女七八个人来到病房，都对着你呵呵地傻笑，谁也不好意思开口问你的病情。只听公司司机小李干咳了两声，很认真地问你：
“听说‘底盘’出了点故障，现在好些了吗？”

你是一名业余作家，一天和朋友一起聊天。
你说：“我们文人的胃口真好，在我们的笔下什么都能吃：吃苦、吃力、吃醋、吃官司、喝西北风、咬文嚼字……还有什么不吃的？”
朋友回答：
“你们不吃软，不吃硬，不吃眼前亏。”

你在专业课表现得很好，就是写论文时间太长。
有一天你自嘲说：“上帝造一个世界只要七天，我写一篇论文怎么要那么长时间？”
同学说：
“你看看上帝造的这个糟糕的世界，再看看你写的这篇优秀的论文……”

你和一个老朋友坐在一家咖啡馆里聊天。
朋友：“你小时候的梦想是什么？”
你：“当警察叔叔。看来我的梦想已经破灭了。”
朋友：
“谁说的？你的梦想已经实现了一半。至少你现在已经成了叔叔。”

阿强在你的店里当伙计。一天早上，他去挂招牌，一不小心，招牌跌成两半。
你很生气地说：“你怎么那么粗心？再这样就走人！”
阿强却不慌不忙地说：
“您别生气，招牌变两半说明您要开分店了，这是很好的预兆啊！”

你是一个跳高能手，但这次运动会没有拿奖。
你看到体育委员后，懊恼地说：“我曾经是跳高冠军呀！你是不是对我很失望？”
他想了一会儿，微笑着说：
“每个班级都想为自己班得冠军而骄傲。如果总是你得冠军，别的班级怎么办？”

你房子后面有一个小菜园，你在菜园里面种蔬菜，可邻居家的鸭子总来偷吃蔬菜。
春节前几天，邻居给你送来一只烤鸭。你对邻居表示感谢。
邻居说：
“谢什么，其实这鸭子是你们家的菜变来的。”

你在自动取款机的小房间熬夜看书。
一个同学发现了就问：“你在这里干什么？”
你说：“看书。”
他又问：“为何要来这里看？”
你还没回答他就自言自语道：
“我知道了，当你看不下去的时候，就把银行卡插进取款机看看余额，就有动力接着看书了。”

你是气象局的一名学生助手。一天，气象学家吩咐你记下星期日的天气预报：早晨晴转多云，午后有大雨。
你一面记一面叹气说：“我周日下午还要去游乐场呢。”
气象学家同情地说：
“那就把大雨划掉吧。”

非攻击非幽默条件

你很擅长跑步。在一次田径比赛中，你因伤跑了最后一名。
你问你的朋友：“我跑了最后一名会不会很没有面子？”
他说道：
“这次你是带伤参赛，发挥有些失常，而你的对手
这次发挥得都很好，所以是情有可原的。”

你走进一家超市，要求购买半棵白菜。
导购员告诉你，超市只出售整棵白菜。
但你坚持只要半棵白菜。
导购员表示，必须请示经理。导购员走进经理办公室说：
“经理，外面有个傻子要买半棵白菜。”
说话时，导购员回过头，发现你站在他身后，
他立刻接着说：
“对不起，我说错了，是这位顾客要买半棵白菜。”

你向你的朋友诉苦：“你不知道我现在的生活过得有多憋屈！当我的妻子发怒时，她会对着我、孩子，甚至对着我们的小狗大喊大叫，而我们谁都不敢吭一声。当我发怒的时候，怎么办呢？”
你的朋友答道：
“那你只好憋在心里，要不就做些剧烈运动缓解情绪。”

朋友：“听说你很怕老师？”

你：“谁说的？我只是某些时候才怕。”
朋友：
“那一定是老师发火的时候吧！”

你是一名胸外科医生。你的车坏了，送到修理部检查，是引擎出了毛病。修理工熟练地把引擎拆下来又装上。
你问修理工：“引擎就是汽车的心脏，我们都是修理心脏的，可是收入为什么差距那么大？”
修理工想了想说：
“因为你们做手术关系到病人的生命。”

你和朋友在大街上看到一群年轻人穿着奇装异服，染着红红绿绿的头发。
你叹了一口气说：“现在的小孩子，内心苍白，只能用外表的时髦来掩饰本质上的土。”
朋友笑着说：
“是啊，真正的时髦还是要从内在的修养做起，修养提高了，外在的气质才会显现出来。”

又过了一个生日，你惆怅自语：“唉，转眼就二十多岁了！”
朋友要安慰你，说：
“所以你要珍惜以后的时间啊！”

你的老板脾气火爆，对你非打即骂。
你有一天实在忍受不了，就问朋友：“这样的老板我怎么能够忍受得了？”
朋友回答说：
“学会忍受是人生的一堂必修课，所以说我们对于生活中无法改变的事实就需要忍受。”

你去买键盘，买完后你又到超市买了两个大铁碗。
你一个朋友正好看到你，就说：
“你一定是不小心把碗打碎了，所以来超市买吧。”

同学问你：“昨夜你寝室有很大的声音，到底什么事？”
你说：“没什么大事，我室友有点不高兴，把我的大衣从楼上扔下去。”
同学：
“声音那么大，你室友至少还扔了一个铁罐下去。”

你和朋友抱怨：“我们家的钱都是我爱人管的，我也太没有地位了！”
朋友说：
“如果你爱人比你更加善于理财，这也是很正常的，不会让你没有地位的。”

上课时，教授请你上台做展示。你在上讲台时没有站稳，差点跌倒。
教授见你稳住身体，对你说：

“这个台阶太高，你上讲台要小心啊！”

你和你的室友吵架后，几天没有说话。这天，室友回到寝室，见你仍在怄气，便不声不响地翻起东西来，把抽屉、衣柜里的衣物全部折腾出来，弄得乱七八糟，满地都是。
你实在忍不住了，问道：“你到底在找什么？”
室友回答说：
“我的手表找不到了，我正在找呢。”

你正在低头赶路，迎面撞上一个人，就道歉说：“对不起对不起，我眼睛瞎了。”
对方笑了笑说：

“你只是走路不小心，没关系的。”

老师把试卷发下来，并要求大家给父母看后请父母在上面签字，再交回来。
第二天，老师问你：“你没有把试卷给父母看吗，为什么没有签字？”
你伸出满是鞭痕的手。
老师说：
“原来他们不肯签字，还打你。”

你上街买了一双挺贵的皮鞋。后来跟同事抱怨说：“都怪我太冲动，一双鞋就花了我半个月工资！”
同事说：
“多亏你只买了一双，要是买两双就是一个月工资了。”

你做了一个痔疮手术，同事们约着一起去医院探望。
男男女女七八个人来到病房，都对着你呵呵地傻笑，谁也不好意思开口问你的病情。
只听公司司机小李干咳了两声，很认真地问你：
“听说你最近不太舒服，现在好些了吗？”

你是一名业余作家，一天和朋友一起聊天。
你说：“我们文人的胃口真好，在我们的笔下什么都能吃：吃苦、吃力、吃醋、吃官司、喝西北风、咬文嚼字……还有什么不吃的？”
朋友回答：
“你说得对，你们的胃口就是很好。”

你在专业课表现得很好，就是写论文时间太长。
有一天你自嘲说：“上帝造一个世界只要七天，我写一篇论文怎么要那么长时间？”
同学说：
“请你不要抱怨，为了保证论文的质量，你需要多花一些时间也是正常的。”

你和一个老朋友坐在一家咖啡馆里聊天。
朋友：“你小时候的梦想是什么？”

你：“当警察叔叔。看来我的梦想已经破灭了。”
朋友：
“谁说的，我刚听说，你已经通过考核，说不定很快就能去警局工作了。”

阿强在你的店里当伙计。一天早上，他去挂招牌，一不小心，招牌跌成两半。
你很生气地说：“你怎么那么粗心？再这样就走人！”
阿强却不慌不忙地说：
“老板，你早该换一个招牌了。这招牌太不结实了。”

你是一个跳高能手，但这次运动会没有拿奖。
你看到体育委员后，懊恼地说：“我曾经是跳高冠军呀！你是不是对我很失望？”
他想了一会儿，微笑着说：
“你忘了？比赛之前你已经病了一个星期，比赛时身体也不舒服，所以成绩肯定受到影响了。下次加油就好！”

你房子后面有一个小菜园，你在菜园里面种蔬菜，可邻居家的鸭子总来偷吃蔬菜。
春节前几天，邻居给你送来一只烤鸭。你对邻居表示感谢。
邻居说：
“谢什么，我家的鸭子老吃你们家的菜，这算是补偿你们的了。”

你在自动取款机的小房间熬夜看书。
一个同学发现了就问：“你在这里干什么？”
你说：“看书。”
他又问：“为何要来这里看？”
你还没回答,他就自言自语道：
“我知道了，今天晚上我们学校宿舍停电了，自习室也关门了，明天又要期末考试，所以你只能在这里看了。”

你是气象局的一名学生助手。一天，气象学家吩咐你记下星期日的天气预报：早晨晴转多云，午后有大雨。
你一面记一面叹气说：“我周日下午还要去游乐场呢。”
气象学家同情地说：
“那就更改去游乐场的日期吧。”
